# Modulation of Protein–Protein Interactions with Molecular Glues in a Synthetic Condensate Platform

**DOI:** 10.1021/jacs.4c17567

**Published:** 2025-01-28

**Authors:** Thijs W. van Veldhuisen, Renske M. J. Dijkstra, Auke A. Koops, Peter J. Cossar, Jan C. M. van Hest, Luc Brunsveld

**Affiliations:** Laboratory of Chemical Biology, Department of Biomedical Engineering and Institute for Complex Molecular Systems, Eindhoven University of Technology, P.O. Box 513, 5600 MB Eindhoven, The Netherlands

## Abstract

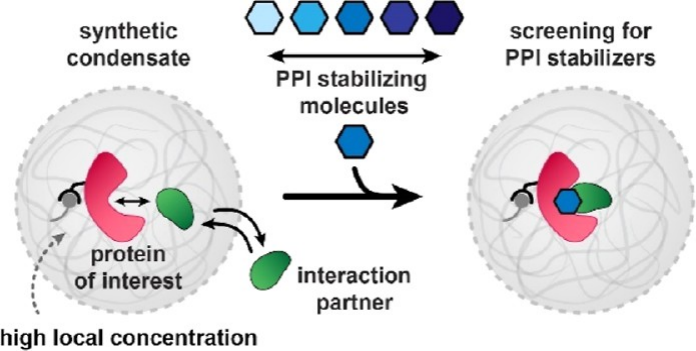

Misregulation of protein–protein interactions (PPIs) underlies many diseases; hence, molecules that stabilize PPIs, known as molecular glues, are promising drug candidates. Identification of novel molecular glues is highly challenging among others because classical biochemical assays in dilute aqueous conditions have limitations for evaluating weak PPIs and their stabilization by molecular glues. This hampers the systematic discovery and evaluation of molecular glues. Here, we present a synthetic condensate platform for the study of PPIs and molecular glues in a crowded macromolecular environment that more closely resembles the dense cellular milieu. With this platform, weak PPIs can be enhanced by sequestration. The condensates, based on amylose derivatives, recruit the hub protein 14-3-3 via affinity-based uptake, which results in high local protein concentrations ideal for the efficient screening of molecular glues. Clients of 14-3-3 are sequestered in the condensates based on their enhanced affinity upon treatment with molecular glues. Fine control over the condensate environment is illustrated by modulating the reactivity of dynamic covalent molecular glues by the adjustment of pH and the redox environment. General applicability of the system for screening of molecular glues is highlighted by using the nuclear receptor PPARγ, which recruits coregulators via an allosteric PPI stabilization mechanism. The condensate environment thus provides a unique dense molecular environment to enhance weak PPIs and enable subsequent evaluation of small-molecule stabilization in a molecular setting chemically en route to the cellular interior.

## Introduction

Protein–protein interactions (PPIs) are vital for the regulation of virtually all cellular processes, from signaling to DNA replication.^1^ Hence, aberrant PPIs are strongly associated with diseases such as cancer^2^ and neurodegenerative diseases,^3^ which has sparked much interest in the development of methods that allow to modulate PPIs with therapeutic agents. Initially, inhibitors of PPIs received significant attention.^1,4−6^ More recently, the stabilization of PPIs via so-called molecular glues has been recognized as an extremely attractive strategy for drug development, which can be used to both activate or inhibit cellular processes.^5,7^ The distinct composite binding interface of PPIs additionally provides unique opportunities for selectivity.^5,7^

Protein–protein interactions are, however, frequently transient and multivalent in nature and often have weak binding characteristics.^8−12^ Such weak PPIs typically show effectiveness via the high local effective molarities created inside cells, such as at membranes, on DNA, or within specific compartments such as biomolecular condensates.^13,14^ Recently, targeting biomolecular condensates with so-called condensate-modifying drugs has emerged as one of the fascinating current developments in the drug discovery field.^15,16^

Notwithstanding the enormous opportunities that molecular glues harbor, there are only a few conceptual approaches for their systematic identification. Next to structural challenges regarding the identification of suitable composite binding pockets,^6^ the transient nature of PPIs and the “three-body-problem” of their stabilization constitute significant barriers for the identification of molecular glues.^6,17^ While structure-based approaches such as fragment-based drug discovery have shown promise for identifying starting points for drug discovery,^18^ high throughput screening approaches have been less successful.^19,20^ Typical assays for ligand screening include fluorescence anisotropy (FA)^6,18^ or time-resolved Förster resonance energy transfer.^6,21^ For a comprehensive overview of ligand screening methods the reader is referred to review articles.^6,22^ During a molecular glue screening campaign involving a weak-affinity PPI, next to molecular diversity, the dilute assay conditions for such screens, are typically not compatible with the high concentrations required for both the PPI binding partners and the compound library members.^6^ Hence, alternative approaches to study and screen molecular glues, which preferably also emulate aspects of the cellular environment, such as molecular crowdedness, are urgently needed to empower molecular glue drug discovery.

Artificial cells are synthetic compartments, typically assembled from synthetic or natural (macro-)molecular building blocks such as polyelectrolytes, polymers, proteins, and lipids, that provide enhanced control over the molecular environment, and more closely resemble cellular environments.^23−28^ In particular, synthetic coacervates or condensates, which are highly concentrated in macromolecules, resemble the crowded environments that are found in the cytosol and cellular compartments such as biomolecular condensates.^23,29^ Artificial cells have, for example, been applied for the replication of a range of biological processes such as the controlled secretion and sensing of macromolecules.^30−33^ We and others have reported on the study of PPIs in such artificial cells^34−38^ and on controlled sequestration of proteinaceous cargo.^39^

Interactions of hub proteins, which are regulatory proteins that influence signaling pathways,^13^ are especially interesting toward molecular glue development.^17^ The 14-3-3 protein is a native hub protein, undergoing a plethora of PPIs important for the regulation of many signaling pathways.^40−43^ 14-3-3 has more than 1200 interaction partners,^44^ of which however most feature only weak PPIs. Interestingly, 14-3-3 has also been implied to regulate targets that form condensates such as the Tau protein.^45,46^ This highlights the need for a controlled platform for molecular glue identification and study of the weak 14-3-3 interactome.

**Figure 1:**
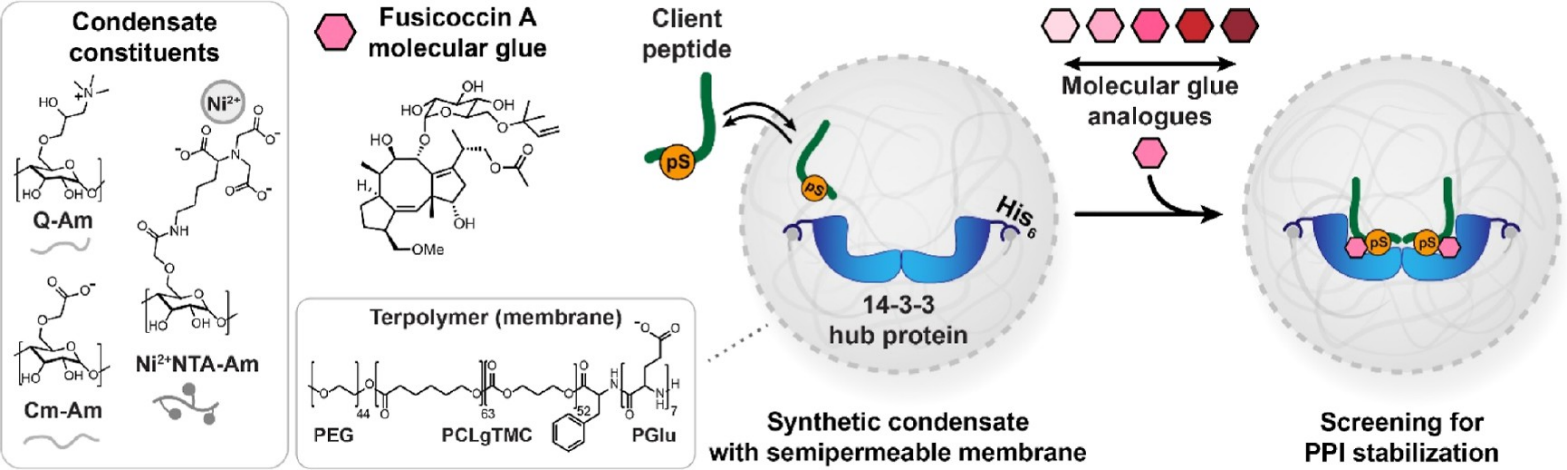
Schematic overview of condensate building blocks and screening of small-molecule PPI stabilization. The constituents include Q-Am and *Cm*-Am, which phase separate to form the condensates, and Ni-NTA-Am, which allows for stable sequestering of His-tagged proteins. The terpolymer forms a stabilizing semipermeable membrane around the condensates. Condensate recruitment of client proteins of the 14-3-3 hub protein can be enhanced by treatment with molecular glues such as Fusicoccin A (3′-deacetylated).

In this work, we describe an artificial cell-mimetic environment based on a synthetic condensate, which enables exploration of small-molecule stabilization of the 14-3-3 hub protein with several of its clients at significantly higher local concentrations than in dilute solution (Figure 1). We successfully demonstrate the stabilization of various 14-3-3-client PPIs within the condensate system with both natural product and (semi)synthetic molecular glues. The high versatility and controllability of the system is shown using the modulation of dynamic covalent molecular glues by changes in pH and redox state. Finally, we illustrate the broad generality of the concept by the ligand-dependent modulation of the PPI of the nuclear receptor peroxisome proliferator-activated receptor gamma (PPARγ) and its coregulators, highlighting the applicability of the system toward a broad spectrum of PPIs.

## Results and Discussion

### Clients of 14-3-3 are Recruited to Condensates upon Treatment with a Molecular Glue

In order to study the molecular glue-enhanced recruitment of clients to condensates, we utilized a synthetic condensate consisting of a mixture of positively charged quaternized amylose (Q-Am, in excess) and negatively charged carboxymethylated amylose (*Cm*-Am), as shown in Figure 1. His-tagged 14-3-3 was recruited to the condensates by interactions with the nitrilo triacetic acid-modified amylose complexed with Ni^2+^ (Ni-NTA-Am). Stabilization of the condensates against fusion was achieved by a semipermeable membrane that is formed by a triblock copolymer (terpolymer).^47^ The His-tagged 14-3-3σ isoform was loaded in the condensates yielding a local 14-3-3 concentration of 16 ± 1 μM as measured by fluorescence quantification, which closely matches the physiological concentration of 14-3-3 in several tissues such as the spleen, large intestine, and prostate (Figure S1 and Table S4).^48^

In a first experiment, several fluorescein isothiocyanate (FITC)-labeled 14-3-3-client peptides, derived from full-length 14-3-3-binding proteins, were selected because of their sensitivity to PPI stabilization by the natural product Fusicoccin A (FC, here referring to 3′-deacetylated FC).^49^ FC is a known molecular glue with efficacies on 14-3-3 interactions in plant and mammalian biology.^50,51^ Cancerous inhibitor of protein phosphatase 2A (CIP2A),^52^ single-stranded DNA-binding protein 4 (SSBP4),^53^ and receptor-interacting Ser/Thr-protein kinase 2 (RIPK2)^53^ are moderate-affinity 14-3-3 binders that bind with their phosphorylated C-terminus to 14-3-3 (Figures S2–S4). These targets were also selected because of their physiological relevance: CIP2A is involved in many types of cancer,^52^ SSBP4 is thought to function as a tumor suppressor,^54^ and RIP2K is involved in inflammatory diseases.^55^

**Figure 2:**
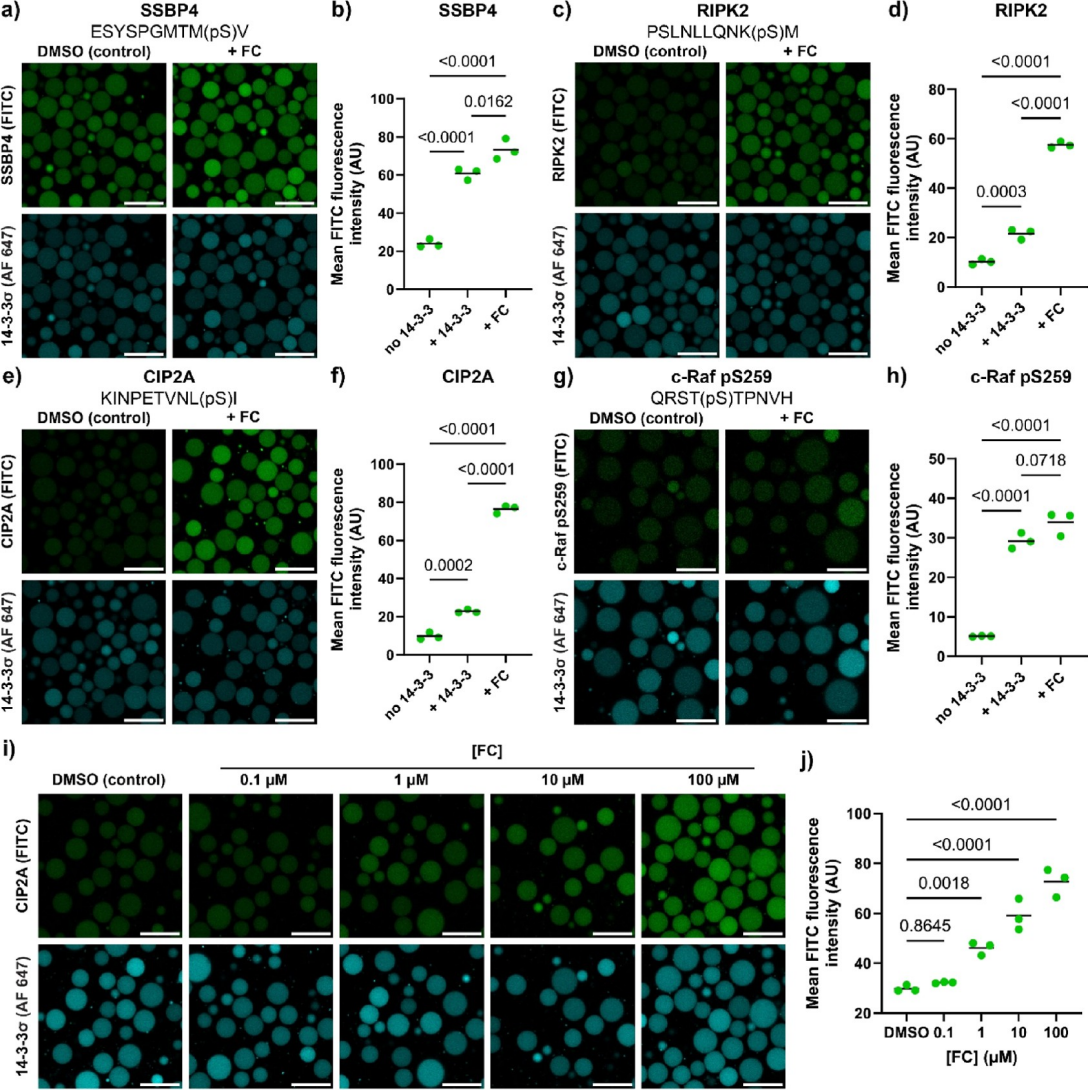
Clients of 14-3-3 are recruited to condensates upon treatment with the molecular glue FC. (a-h) Confocal micrographs of 14-3-3σ-loaded condensates supplied with client peptides (100 nM) in the absence (DMSO control) or presence of 100 μM FC, including quantification. The samples were incubated overnight. Scale bar: 25 μm. The micrographs of samples without 14-3-3σ are shown in Figure S5. Statistical analysis was performed by one-way ANOVA with Tukey’s test with correction for multiple comparisons, with *N* ≥ 49 condensates across 2 imaging positions in 3 independent samples. *P* values are shown above the comparison, with significance regarded as *P* < 0.05. The line shows the mean, and the symbols show the individual measurements. (i) Confocal micrographs of 14-3-3σ-loaded condensates supplied with the CIP2A peptide (100 nM) in the absence (DMSO control) or presence of various concentrations of FC, incubated overnight. Scale bar: 25 μm. (j) Quantification of micrographs of 14-3-3σ-loaded condensates supplied with the CIP2A peptide (100 nM) in the absence (DMSO control) or presence of various concentrations of FC. Statistical analysis was performed by one-way ANOVA with Dunnet’s test with correction for multiple comparisons, compared to the DMSO control. *N* ≥ 55 condensates across 2 imaging positions in 3 independent samples. *P* values are shown above the comparison, with significance regarded as *P* < 0.05. The line shows the mean and the symbols show the individual measurements.

Loading of the condensates with 14-3-3 induced recruitment of the client peptides as compared to condensates without 14-3-3 (Figures 2a–f and S5). All three FC-dependent 14-3-3 client peptides showed a strongly enhanced recruitment into the condensates upon treatment with FC, as analyzed by confocal microscopy (Figure 2a–f). c-Raf pS259,^56^ a control peptide that also binds to 14-3-3 but is not stabilized by FC, did not show a significant condensate recruitment in response to the molecular glue (Figures 2g,h and S6).

The SSBP4 peptide is the intrinsically strongest binding FC-responsive client (Figure S3). This high natural affinity resulted in the smallest enhancement in recruitment upon treatment with the molecular glue (1.2-fold, Figure 2b). With a 10-fold reduction in the concentrations of both binding partners 14-3-3σ and SSBP4, the PPI became more sensitive to FC with a 1.9-fold change in SSBP4 recruitment, highlighting the system’s capability for tuning the window of molecular modulation of the stabilization of the PPI at hand (Figure S7) and that the platform is especially suited to study stabilization of weak PPIs.

Another means of modulating the assay window of the system is by adjusting the formulation of the condensates. The CIP2A peptide has a net negative charge (−4) at neutral pH. When the condensate formulation was adjusted to a more positively charged one, the partitioning of the peptide based on electrostatic interactions reached higher levels (Figure S8). In contrast, a more neutral condensate formulation generated a greater fold change in peptide recruitment due to a low degree of background partitioning of the peptide. This demonstrates that the condensate formulation can be used to improve the assay window of PPI stabilization. These results thus showed that synthetic condensates can be used to induce and study 14-3-3-based PPI stabilization with molecular glues.

### Molecular Glue-Induced Client Recruitment is Dose-Dependent

Next, we studied 14-3-3 client recruitment into the condensates as a function of FC molecular glue concentration (Figure 2i,j). Starting from an FC concentration of 1 μM (10 eq. relative to 14-3-3 and CIP2A), a significant dose-dependent effect on CIP2A client recruitment was observed. To determine if partitioning of the molecular glues between the bulk and condensate phase could affect the PPI stabilization within the condensate,^57−59^ we developed a liquid chromatography coupled to mass spectrometry (LC–MS) assay to quantify the partitioning of FC (Figure S9). With this assay, the partitioning coefficient of FC to the condensate phase was determined to be 0.96 ± 0.02. This means that the FC concentration inside the condensates was similar to that in bulk solution.

### Screening of a Panel of Molecular Glues Yields Differentiated Client Recruitment

**Figure 3:**
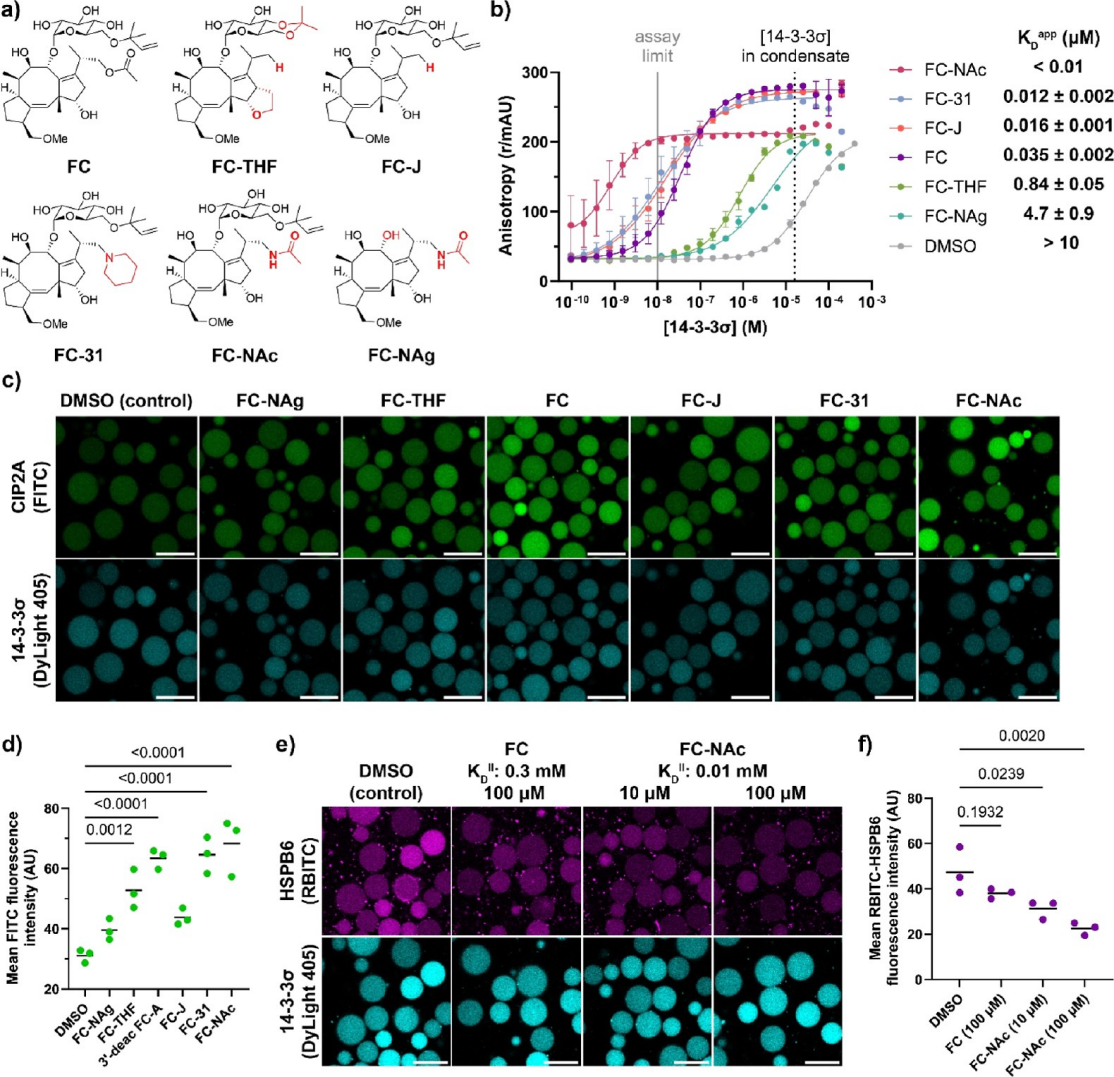
Screening of a panel of molecular glues yields differentiated client recruitment. (a) Structures of selected FC analogues. The differences are highlighted in red. (b) Protein titration of 14-3-3σ to the CIP2A peptide as measured by FA assay in the presence of DMSO (control) or 100 μM of various FC analogues. Symbols represent the mean of a triplicate measurement, with the error bars partly obscured by the symbols. Lines show fits by a 4-parameter logistic regression model. The resulting *K*_D_ obtained from the fit is shown as mean with the standard error. The dotted line shows the local [14-3-3σ] inside the condensates, and the gray line shows the assay limit (10 nM). (c) Confocal micrographs of 14-3-3σ-loaded condensates supplied with CIP2A (100 nM) in the absence (DMSO control) or presence of 1 μM of FC analogues. Samples were incubated overnight. Scale bar: 25 μm. (d) Quantification of micrographs of 14-3-3σ-loaded condensates and CIP2A (100 nM) in the absence (DMSO control) or presence of 1 μM of FC analogues. Statistical analysis was performed by one-way ANOVA with Dunnett’s test with correction for multiple comparisons to the DMSO control, with *N* ≥ 89 condensates across 2 imaging positions in 3 independent samples. *P* values are shown above the comparison, with significance regarded as *P* < 0.05. The line shows the mean and the symbols show the individual measurements. (e) Confocal micrographs of 14-3-3σ-loaded condensates supplied with HSPB6 (150 nM) in the absence (DMSO control) or presence of FC analogues. Samples were incubated overnight. Scale bar: 25 μm. (f) Quantification of micrographs of 14-3-3σ-loaded condensates and HSPB6 (100 nM) in the absence (DMSO control) or presence of FC analogues. Statistical analysis was performed by one-way ANOVA with Tukey’s test with correction for multiple comparisons, with *N* ≥ 52 condensates across 2 imaging positions in 3 independent samples. *P* values are shown above the comparison, with significance regarded as *P* < 0.05. The line shows the mean, and the symbols show the individual measurements.

After demonstrating the FC-dependent recruitment of several 14-3-3 clients, we sought to screen a specific 14-3-3 PPI against several molecular glues with varying stabilizing efficacy (Figure 3). We selected a panel of semisynthetic FC analogues with varying stabilization factors (SF),^60^ defined as the fold difference between the binary K_D_ and the apparent *K*_D_ in the presence of a compound (*K*_D_^app^). The 14-3-3σ/CIP2A interaction was screened against six FC analogues (Figure 3a,b). At 1 μM, only the FC and FC-NAc molecular glues showed significant enhancement of recruitment of CIP2A, as analyzed by confocal microscopy (Figure S10). These molecular glues are also among the best performing ones in a traditional FA assay (Figure 3b). By increasing the molecular glue concentration to 100 μM, the molecular glues FC-THF and FC-31 also showed significant enhancement of CIP2A recruitment (Figure 3c,d). Only molecular glue FC-J showed a differentiated performance between the FA assay and the condensates.

### Condensate Environment Influences Competitive Binding Events

Competitive binding of multiple clients to 14-3-3 can be modulated by molecular glues when the molecular glue possesses PPI selectivity.^61^ In addition, there can also be competition between a client and a molecular glue for 14-3-3 binding: the interaction of 14-3-3σ with heat shock protein beta-6 (HSPB6)^62^ is of moderate affinity (*K*_D_: 0.99 ± 0.03 μM, Figure S11) and the binding mode of this client overlaps with the binding pocket of FC derivatives (Figure S12). We assessed the responsivity of this PPI toward FC and FC-NAc in the condensates. FC-NAc has a higher intrinsic affinity for apo 14-3-3σ (K_D_^II^: 0.01 mM) than FC (K_D_^II^: 0.3 mM) (Figure S11),^63^ and indeed only FC-NAc featured significant competition with HSPB6 for 14-3-3 binding, resulting in HSPB6 release from the condensates (Figure 3e,f).

Next, the competitive binding of 14-3-3 clients, illustrated by HSBP6 and CIP2A, was analyzed. Both peptides were added to 14-3-3σ-loaded condensates, each at a 1.5-fold molar excess relative to 14-3-3σ. Interestingly, CIP2A outcompeted the majority of HSPB6 even in the absence of molecular glues (Figure S13), although in dilute solution HSBP6 binds to 14-3-3σ with a >10-fold lower K_D_. This differentiated PPI behavior within condensates compared to conventional solution assays might have resulted from intrinsic condensate affinity related to peptide charge. HSPB6 is charge-neutral at neutral pH, whereas CIP2A has a net charge of −3 that favors partitioning to the positively charged condensates (Table S1 and Figure S13d–g).^37^ This observation indicates how coacervate environments can modulate competitive PPI binding events and provides a path to mimic differentiating cellular conditions for PPIs.

### Split Luciferase Assay Enables Protein–Protein Interaction Screening without Changes in Localization

**Figure 4:**
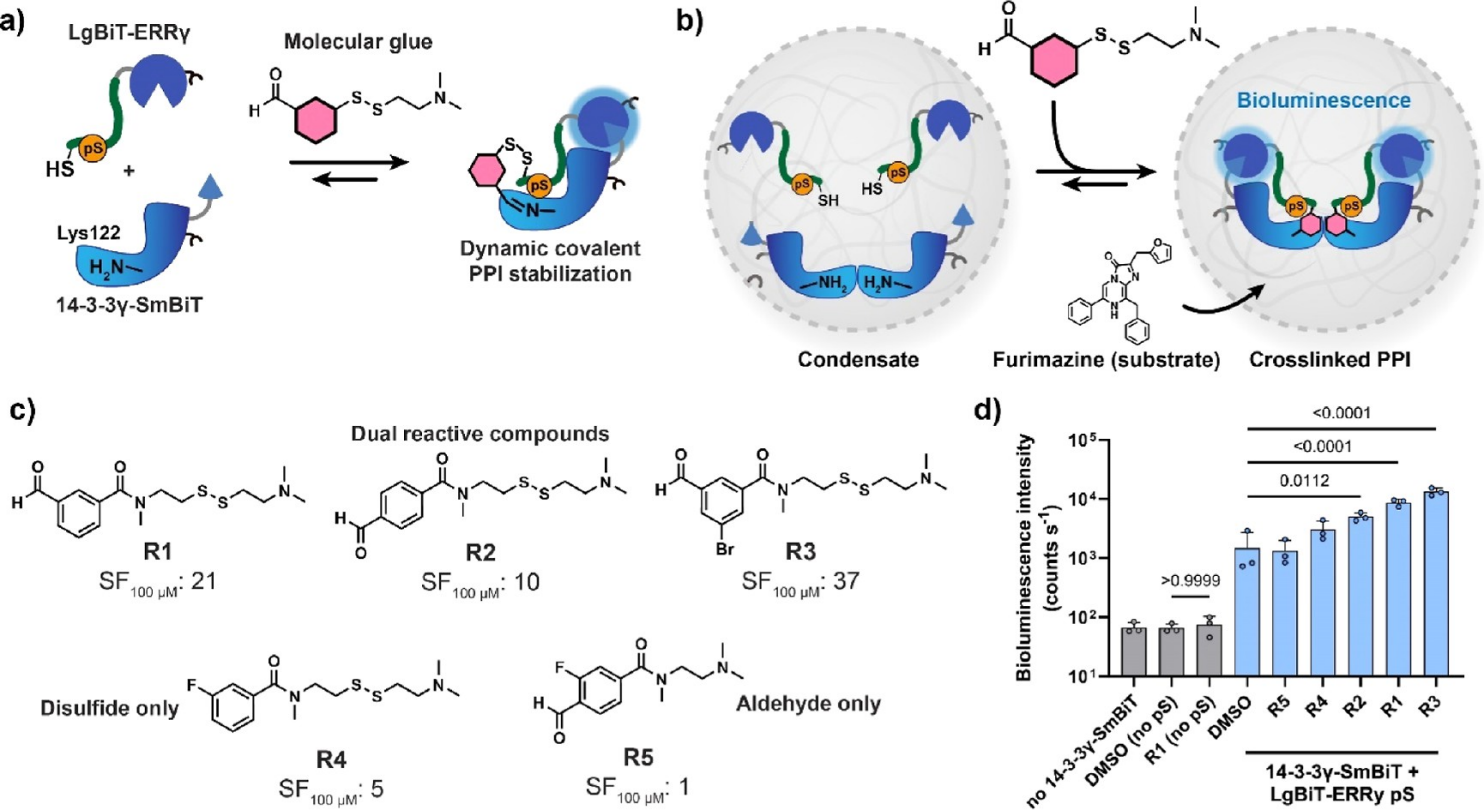
Screening of molecular glues inside condensates by a split luciferase assay. (a) Schematic overview of split luciferase assay for the 14-3-3γ/ERRγ interaction, stabilized by reactive molecular glues. (b) Assay setup of the split luciferase assay inside condensates. (c) Panel of reactive molecular glues for the 14-3-3γ/ERRγ interaction, with stabilization factor at 100 μM compound concentration (SF_100μM_) shown, as determined in ref (61) (d) Split luciferase assay for the dynamic covalent stabilization of the 14-3-3γ/ERRγ interaction inside condensates. Assay conditions: 250 pM of LgBiT-ERRγ, phosphorylated or lacking the phosphorylation site (no pS), 250 pM of 14-3-3γ-SmBiT, 100 μM of reactive compounds **R1–R5**, 50 μM of TCEP, and 1 v/v % of DMSO. The samples were incubated overnight, after which the substrate furimazine was added (1:1000 dilution) and the samples were measured immediately. Statistical analysis was performed by one-way ANOVA with Tukey’s test with correction for multiple comparisons, with *N* = 3 independent condensate samples. *P* values are shown above the comparison. The bar plot shows the mean ± standard deviation, and the symbols show the individual measurements.

We also developed an assay format where protein partners can be prelocalized to the condensates by His-tags. In this plate reader assay format, we study, as example, the 14-3-3γ/ERRγ interaction in condensates (Figure 4a).^61^ Here, we opted for a split luciferase assay to analyze the stabilization of the PPI. The large part of split NanoLuc,^64^ LgBiT, was fused to the ERRγ domain, and the small part, SmBiT, was fused to 14-3-3γ. Both fusion proteins were anchored in the condensates by His-tag interactions with Ni-NTA-Am (Figure 4b). Upon an increased proximity of the luciferase subunits by PPI stabilization, an enhanced bioluminescence signal was expected. The phosphorylated LgBiT-ERRγ indeed showed an enhanced bioluminescence signal compared to its unphosphorylated form due to its affinity for 14-3-3γ.

We selected a panel of five molecular glues with SF_100μM_ values of 1 to 37^61^ (Figure 4c) as early stage hit matter for drug discovery. The molecular glues in this case had dynamic covalent mechanisms of action, forming both an imine bond with Lys122 of 14-3-3γ and a disulfide bond with a Cys residue of ERRγ. 14-3-3γ and ERRγ were loaded in the condensates at 250 pM, and the samples were analyzed after overnight incubation in the presence of 100 μM of molecular glue (Figures 4d and S14). Satisfyingly, the dual covalent molecular glues **R1**, **R2**, and **R3** all induced a significantly enhanced bioluminescence signal, higher than the mono reactive **R4** molecular glue. The control reactive molecular glue **R5**, which does not stabilize this PPI in solution, also produced no change in the bioluminescence within the condensates. The nonphosphorylated LgBiT-ERRγ (no pS) was unresponsive to the molecular glue stabilization, testifying to the on-mechanism character of the observed PPI stabilization.

### Condensates Have an Adjustable Environment that Empowers Screening under Specific Conditions

Various cellular compartments such as biomolecular condensates have characteristic local physicochemical environments, for example, with unique pH values.^65^ Here, we sought to demonstrate the adjustability of the condensates toward specific environments using the 14-3-3γ/ERRγ dual covalent molecular glues. Importantly, the formation of the molecular glue-cross-linked PPI in bulk solution was shown to be dependent on both the pH (Figure S15 and Table S5) and the reduction/oxidation state.^61^

First, using the ratiometric pH sensor protein mCherryEA^66^ inside the condensates, we showed that the local pH values inside the condensates closely matched the pH in bulk solution across the pH range from 6.5 to 8.5 (Figure S16).

**Figure 5:**
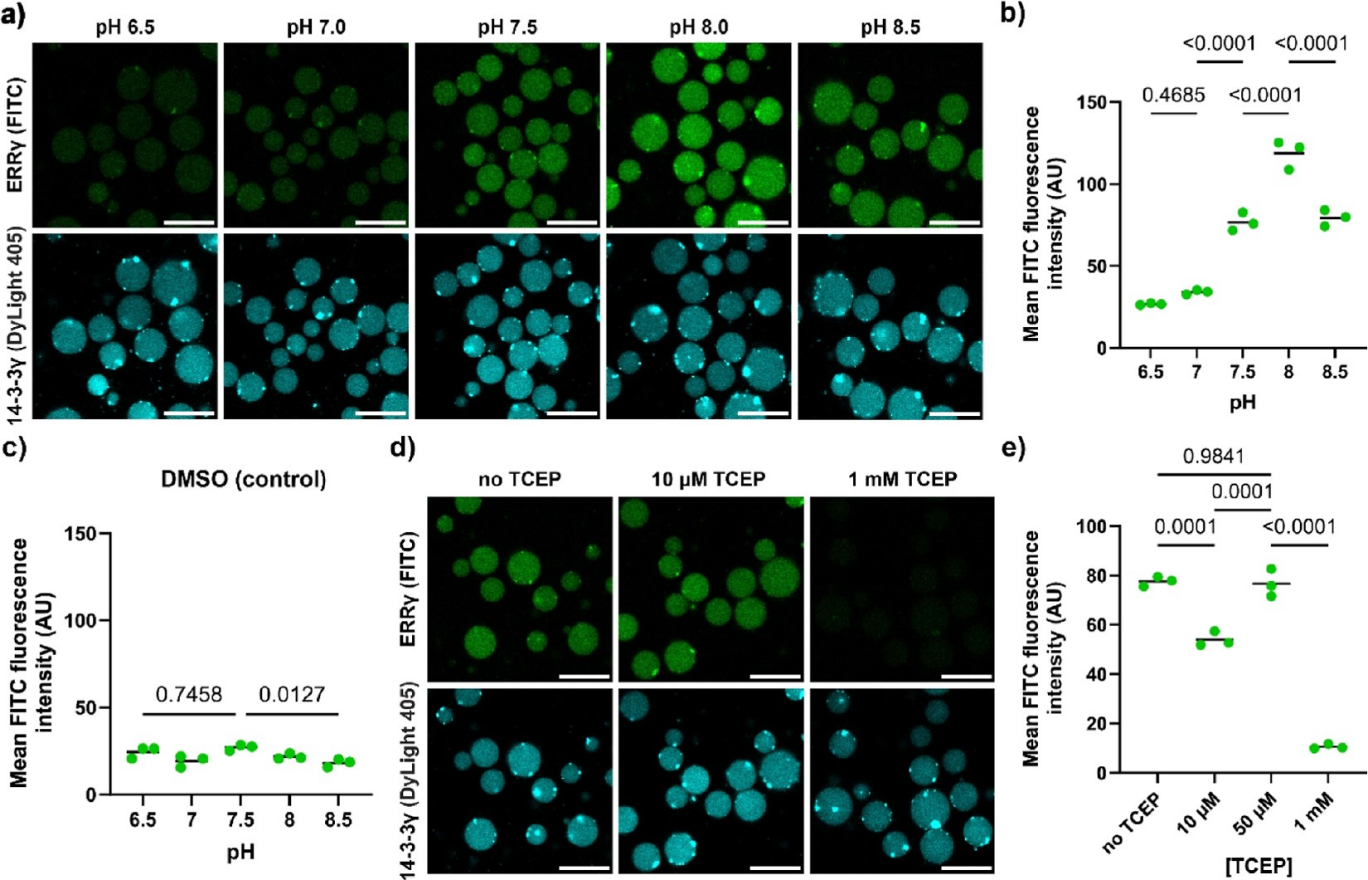
Condensate environment provides adjustability for screening under specific conditions. (a) Confocal micrographs of 14-3-3γ-loaded condensates supplied with FITC-ERRγ (100 nM) in the presence of 100 μM of stabilizer **R1** at various pH values and in the presence of 50 μM of TCEP. Samples were incubated overnight. Scale bar: 25 μm. The 14-3-3γ isoform had a tendency to show aggregation. The coinciding increased signal intensity in the ERRγ channel indicates that the PPI formation was for the majority not affected. A limited number of aggregates seem to not be available for ERRγ binding, although differences in fluorophore signal intensity also play a role in these observations. (b,c) Quantification of micrographs of 14-3-3γ-loaded condensates supplied with FITC-ERRγ (100 nM) in the presence of 100 μM of stabilizer **R1** (b) or DMSO (c) at various pH values. Statistical analysis was performed by one-way ANOVA with Tukey’s test with correction for multiple comparisons, with *N* ≥ 47 condensates across 2 imaging positions in 3 independent samples. *P* values are shown above the comparison, with significance regarded as *P* < 0.05. The line shows the mean, and the symbols show the individual measurements. Confocal micrographs of samples used for panel c are available in Figure S17. (d) Confocal micrographs of 14-3-3γ-loaded condensates supplied with FITC-ERRγ (100 nM) in the presence of 100 μM of stabilizer **R1** at various TCEP concentrations and pH 7.5. Samples were incubated overnight. Scale bar: 25 μm. (e) Quantification of micrographs of 14-3-3γ-loaded condensates supplied with FITC-ERRγ (100 nM) in the presence of 100 μM of stabilizer at various TCEP concentrations. Statistical analysis was performed by one-way ANOVA with Tukey’s test with correction for multiple comparisons, with *N* ≥ 37 condensates across 2 imaging positions in 3 independent samples. *P* values are shown above the comparison, with significance regarded as *P* < 0.05. The line shows the mean, and the symbols show the individual measurements.

The FITC-labeled ERRγ peptide was subsequently added to condensates with 14-3-3γ. The binary 14-3-3γ/ERRγ interaction was mostly pH-insensitive within the condensates (Figures 5c and S17). However, in the presence of aldehyde-functional **R1** an enhanced recruitment at higher pH values could be observed, reflecting the improved reactivity of the molecular glues (Figure 5a,b). Interestingly, the condensate recruitment of ERRγ was lower at pH 8.5 compared with pH 8.0. Possibly, elevated pH values drove off-target reactivity toward other Lys residues in the condensates, which was not observed in dilute solution. This could have been mediated by the crowded condensate environment and high local concentrations of interaction partners as these molecular glues were generally specific.^61^ Such off-target reactivity might also be correlated to off-target effects in cellular environments.

In a subsequent experiment tris(2-carboxyethyl)phosphine (TCEP) was used as catalyst for disulfide exchange between the molecular glues and the ERRγ client. First, TCEP was shown to not be enriched in the positively charged condensates, using the ratiometric FROG/B sensor protein^67^ (Figure S18). The 14-3-3/ERRγ PPI stabilization by the dynamic covalent molecular glues in the condensates was halted at high concentrations of TCEP (Figure 5d,e). These observations align with the observations in bulk solution^61^ and reflect the absence of disulfide formation under these condensate conditions.

Finally, we confirmed that the formation of the dual covalent cross-linked PPI by the molecular glues occurred on a similar time scale as in the solution (Figure S19). This indicates that stabilization of the PPI within the condensates by the molecular glues is not the result of enhanced local reactivity of the molecular glues.

### Molecular Glue Stabilization of a Nuclear Receptor PPI Shows the Broad Applicability of the Condensate System

A different PPI, not involving 14-3-3, was studied to investigate the general applicability of the condensate system for screening small-molecule modulators of PPIs. The nuclear receptor PPARγ regulates fatty acid storage and glucose metabolism and is involved in diseases such as cancer and diabetes.^68,69^ Natural and synthetic PPARγ ligands have found clinical use,^70^ and a range of partial as well as full agonists have been reported.^71^

**Figure 6:**
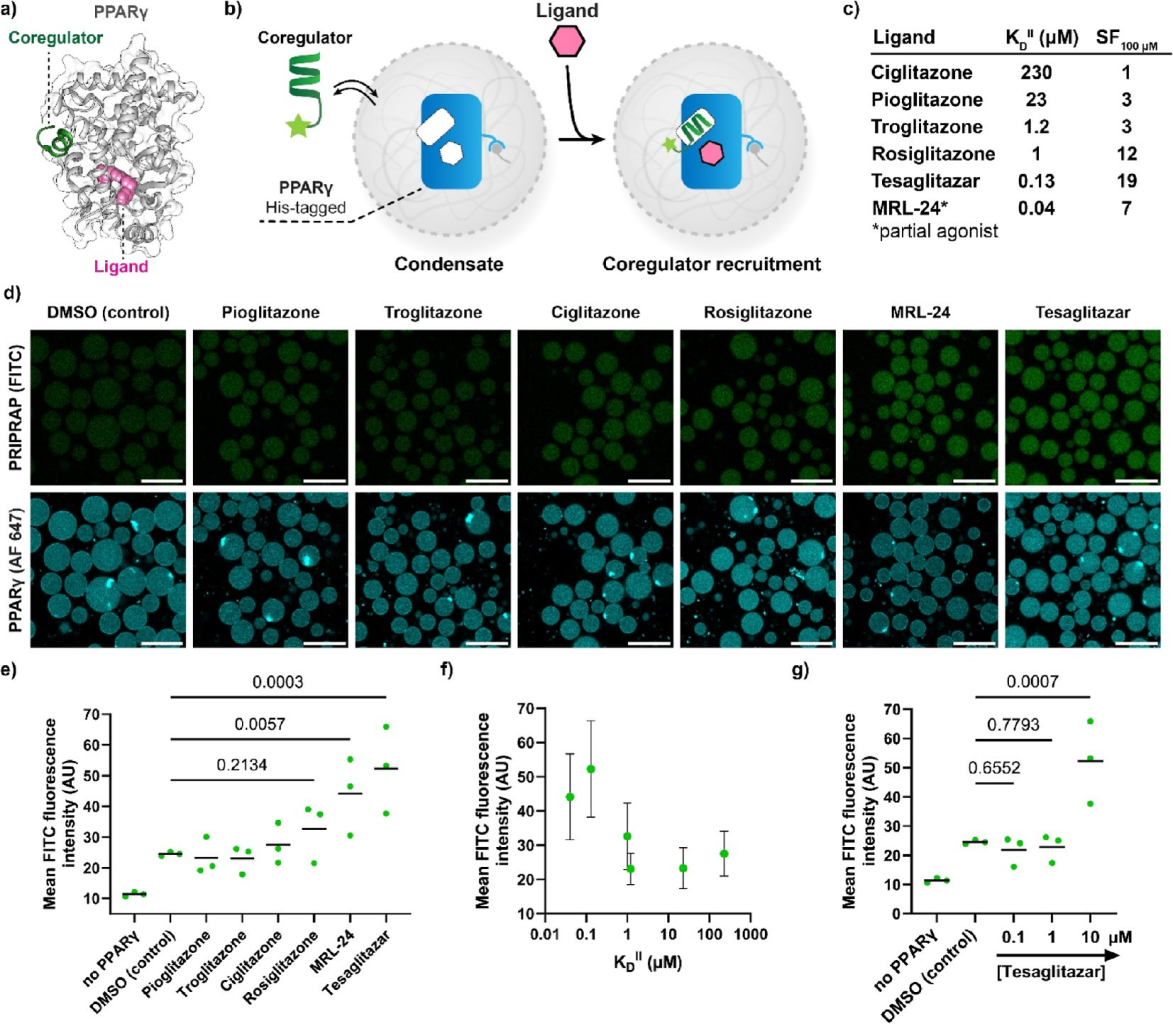
PPIs of the nuclear receptor PPARγ with coregulators can be modulated with ligands inside condensates. (a) Crystal structure of the ligand binding domain of PPARγ with bound coregulator peptide PRIPRAP and ligand rosiglitazone, PDB: 6ONJ.^74^ (b) Schematic overview of ligand-dependent recruitment of coregulators in condensates loaded with PPARγ. (c) Overview of commercial PPARγ ligands, with intrinsic affinity *K*_D_^II^ as determined by ref (73) The stabilization factor was calculated by the fold change in *K*_D_^app^ in the presence of the compounds. (d) Confocal micrographs of ligand-dependent recruitment of PRIPRAP coregulator (100 nM). Ligands were added at a concentration of 10 μM. Samples were incubated overnight. Scale bar: 25 μm. (e,f) Quantification of PRIPRAP from micrographs taken at the conditions of panel (d). Data was plotted per ligand (e) and against the *K*_D_^II^ of the ligands (f). Statistical analysis was performed by one-way ANOVA with Tukey’s test with correction for multiple comparisons, with *N* ≥ 46 condensates across 2 imaging positions in 3 independent samples. *P* values are shown above the comparison, with significance regarded as *P* < 0.05. The line shows the mean, and the symbols show the individual measurements. (g) Quantification of micrographs of PPARγ-loaded condensates supplied with PRIPRAP (100 nM) at various concentrations of tesaglitazar. Statistical analysis was performed by one-way ANOVA with Dunnet’s test with correction for multiple comparisons, compared to the DMSO control. *N* ≥ 46 condensates across 2 imaging positions in 3 independent samples. *P* values are shown above the comparison, with significance regarded as *P* < 0.05. The line shows the mean, and the symbols show the individual measurements. The micrographs are available in Figure S25.

Ligand binding to PPARγ induces conformational changes, which then leads to changes in coregulator binding affinities (Figure 6a).^72^ Due to its therapeutic relevance and its allosteric mode of PPI stabilization, we selected PPARγ as a model system for the illustration of the condensate system toward a broad range of druggable PPIs.

His-tagged PPARγ (400 nM) was loaded into the condensates (Figure 6b), yielding a local PPARγ concentration of 2.0 ± 0.4 μM (Figure S20). We selected a panel of commercially available PPAR ligands (Figure 6c) with varying intrinsic affinity for PPARγ and cooperativity for coregulator binding (Figure S21).^73^ The ligands were added to the PPARγ-loaded condensates at a ligand concentration of 10 μM, while monitoring the recruitment of the coregulator peptide PRIPRAP^75^ into the condensates by confocal microscopy (Figure 6d,e). The intrinsic affinity of the ligand for PPARγ, *K*_D_^II^, favorably correlated with the coregulator recruitment to the condensates (Figure 6f). The ligands MRL24 (partial agonist) and tesaglitazar (full agonist) both induced significant recruitment of PRIPRAP relative to the DMSO control. It was found that the condensate formulation led to the observed variability in the data, as the experiments were performed in independent triplicate (Figure S22).

We used the autofluorescent PPARγ ligand chiglitazar, omitted from the original ligand panel due to its autofluorescence, to probe the partitioning coefficients of such ligands. Chiglitazar contains a carboxylic acid like most other PPARγ ligands and is similar in log*P* to MRL24 (6.8 vs 6.9, respectively). The partitioning coefficient for chiglitazar was determined to be 23 ± 4 (Figure S23), indicating that such ligands are enriched in the condensate by interactions with the positively charged condensate environment. Similarly, MRL24 was potentially enriched, explaining its relatively strong effect on the coregulator recruitment despite being a partial agonist.

The dose dependence of the PPI formation was studied using tesaglitazar (Figures 6g, S24 and S25). Here, we found a significant effect in PPI stabilization in the condensates at 10 μM compound. We validated the tesaglitazar-dependent recruitment of coregulators with two additional coregulators that bind PPARγ with either high (MED1 DRIP-2, *K*_D_ 647 ± 62 nM) or low affinity (EP300, *K*_D_ > 10 μM) (Figures S26 and S27). Also for these two coregulators, a ligand-mediated stabilization of the PPI was observed in the condensates, testifying to the wide range of coregulators that can be studied with this system.

**Figure 7:**
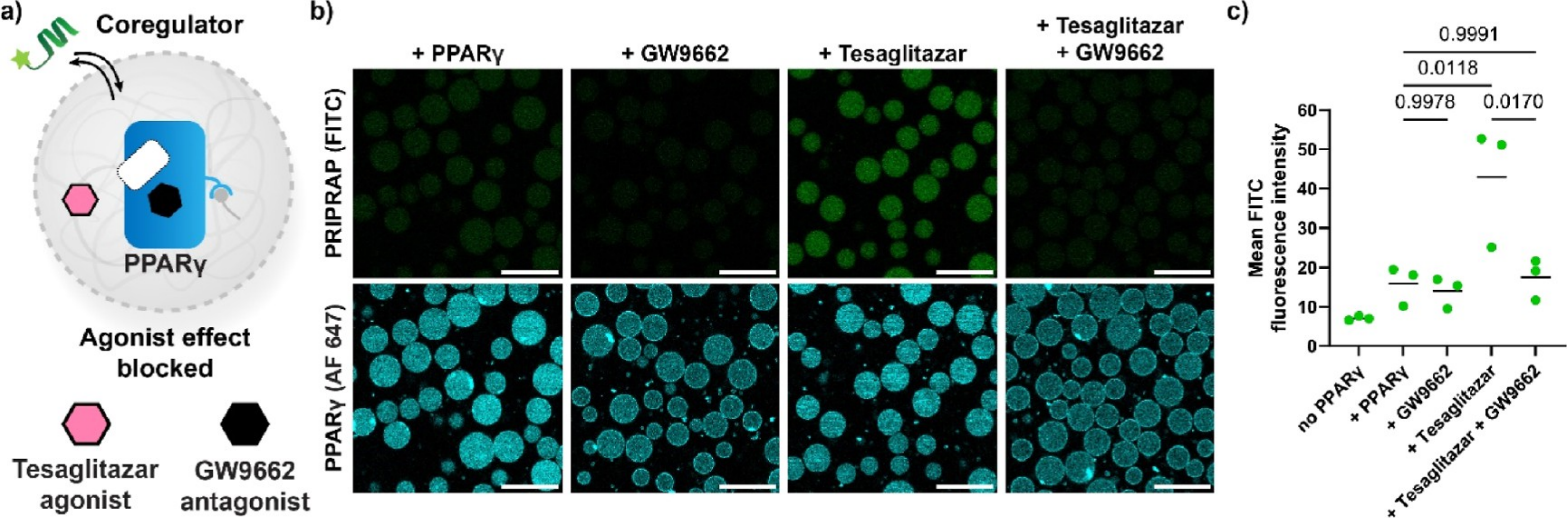
Antagonist GW9662 blocks the effect of ligands on coregulator recruitment to the condensates. (a) Schematic overview of coregulator recruitment into PPARγ-loaded condensates upon treatment with agonist and/or antagonist. (b) Confocal micrographs of condensates loaded with PPARγ (400 nM) and in the presence of coregulator PRIPRAP (100 nM), treated with tesaglitazar (agonist, 10 μM) and/or GW9662 (antagonist, 10 μM). Samples were incubated overnight. Scale bar: 25 μm. (c) Quantification of confocal micrographs showing PPARγ-, agonist-, and/or antagonist-dependent recruitment of the coregulator PRIPRAP. Statistical differences were analyzed by Tukey’s test with correction for multiple comparisons, with *N* ≥ 132 condensates across 2 imaging positions in 3 independent samples. *P* values are shown above the comparisons, with significance regarded as *P* < 0.05. Solid lines represent the mean, and the symbols represent individual measurements.

As a final test-case, we sought to demonstrate that the condensate molecular glue system is also applicable to screen interactions of competing ligands. GW9662 is a PPARγ antagonist that blocks agonist binding by covalently anchoring in the ligand binding pocket.^76,77^ Condensates were prepared with PPARγ, and the coregulator PRIPRAP was added in the presence of DMSO (control), GW9662 (10 μM), tesaglitazar (10 μM), or both compounds (Figure 7a).

GW9662 itself did not influence PRIPRAP recruitment, as expected (Figure 7b,c). In codosing with tesaglitazar, the antagonist convincingly inhibited the stabilizing effect of tesaglitazar, with the PRIPRAP recruitment at a similar level as the DMSO control. The condensate system is thus also suitable for the analysis of ligand competition.

## Conclusions

A synthetic condensate platform was developed to enable the study of molecular glue stabilization of PPIs in up-concentrated dense molecular environments. This addresses the urgent need to be able to screen and study weak PPIs and their stabilization with molecular glues. PPIs of the clinically relevant 14-3-3 hub protein and of the PPARγ nuclear receptor could both be stabilized in the condensates with molecular glues in a dose-dependent and efficacy-dependent manner. Convincingly, 14-3-3 clients that were not responsive to the natural product FC in conventional solution-based assays also yielded no response in the condensates or could even be displaced by virtue of the molecular glue. The dynamic range of the assay could be adjusted by modulation of the coacervate formulation, thereby changing the partitioning of the components. The system is applicable to low- or medium-affinity PPIs, as the elevated concentrations in the condensate already lead to complex formation.

The enhanced partitioning of selective molecular glues into the synthetic condensates, such as shown for the PPARγ ligands, bears resemblance to native biomolecular condensates^57,59^ and also provides further opportunities of this synthetic platform toward evaluation of condensate-modifying drugs.^15,16^ Hence, the synthetic condensate platform enables ligand screening at high local concentrations of interaction partners in a crowded environment, which is typically not possible with the current in vitro assays.

Next to microscopic evaluation, we also developed a plate reader assay for the evaluation of molecular glues in condensates more compatible with classical high-throughput screening. Split luciferase complementation via stabilization of the 14-3-3γ/ERRγ PPI also correlated strongly with the stabilization factor determined for the dynamic covalent molecular glues, as determined by conventional assays in dilute solution. This luminescence-based condensate system also allowed us to highlight the straightforward modulation of environmental parameters of the synthetic condensates, such as pH and redox potential, to potentially match specific biological environments like the cytoplasm or cellular compartments.

The general applicability of the condensate system for PPI molecular glue screening was highlighted using small-molecule stabilization of coregulator binding of the nuclear receptor PPARγ. We validated the ligand-dependent recruitment of a set of coregulators and were able to show a correlation with intrinsic affinity of the ligands with recruitment of the PRIPRAP coregulator to the synthetic condensates. Convincingly, an antagonist was able to prevent ligand-based coregulator recruitment to the condensates. This shows that more complex protein targets with allosteric molecular glue mechanisms and defined ligand binding pockets can also be screened in this system.

The modulation of PPIs with molecular glues in synthetic condensates provides a novel and unique platform to both screen and study the stabilization of weak PPIs and to study PPI stabilization with molecular glues in crowded molecular environments, two challenges that have been typically ignored thus far. This novel platform enables the execution of drug discovery assays in a controlled, crowded environment. This makes it complementary and bridges the dilute biochemical assays and complex cellular studies that are typical in drug discovery pipelines. The platform can logically be extended toward other PPIs and different condensate environments and to include more complex up- or downstream signaling events. We also envision the replacement of the amylose-derived polyelectrolytes with proteinaceous and nucleic acid building blocks to more accurately capture the diverse noncovalent interactions that occur within cellular environments and biomolecular condensates.

## Supporting Information Available

Detailed experimental procedures, Figures S1-S40, and Tables S1-S5: quantification of the concentration of 14-3-3σ inside the condensates; protein titration of 14-3-3σ to the CIP2A peptide, SSBP4 peptide, and RIP2K peptide; confocal micrographs of condensates; protein titration of 14-3-3σ to the non-FC-responsive c-Raf pS259 peptide; crystal structure of 14-3-3σ/CIP2A interaction in the presence of Fusicoccin A; brightfield micrograph of a representative condensate sample for the split luciferase ERRγ assay in condensates; time-dependent and pH-dependent protein titration of 14-3-3γ to 100 nM of FAM-labeled ERRγ peptide; schematic overview of mCherryEA structure and uptake into synthetic condensates; binary 14-3-3/ERRy interaction is mostly non-sensitive to pH; measurement of the local concentration of PPARγ in the condensates; protein titrations of PPARγ to the PRIPRAP peptide; quantification of PRIPRAP signal from condensates; absorbance spectrum of chiglitazar; LC-MS Q-ToF chromatograms; m/z spectra; and ^1^H NMR spectra (PDF)

## Abbreviations

CIP2A: cancerous inhibitor of protein phosphatase 2A
FA: fluorescence anisotropy
FC: Fusicoccin A
FITC: fluorescein isothiocyanate
HSPB6: heat shock protein beta-6
LC-MS: liquid chromatography coupled to mass spectrometry
Ni-NTA-Am: nitrilo triacetic acid-modified amylose complexed with Ni^2+^
PPARγ: peroxisome proliferator-activated receptor gamma
PPI: protein–protein interaction
Q-Am: quaternized amylose
RIPK2: receptor-interacting Ser/Thr-protein kinase 2
SF: stabilization factor
SSBP4: single-stranded DNA-binding protein 4
TCEP: tris(2-carboxyethyl)phosphine
