# Supplementary material for: Modulation of Protein–Protein Interactions with Molecular Glues in a Synthetic Condensate Platform
Source: J Am Chem Soc. 2025 Jan 28;147(6):5386–97. doi: 10.1021/jacs.4c17567 (PMC11826995; doi:10.1021/jacs.4c17567)
Supplement: Supplementary file 1 — ja4c17567_si_001.pdf [file ja4c17567_si_001.pdf]

Supplementary Information

Modulation of protein-protein interactions with molecular glues in a synthetic condensate platform

Thijs W. van Veldhuisen, Renske M. J. Dijkstra, Auke A. Koops, Peter J. Cossar, Jan C. M. van Hest*, Luc Brunsveld*

Experimental Section

Materials and instruments

14-3-3σ and 14-3-3γ were kindly provided by Maxime van den Oetelaar, Carlo Verhoef, and Marloes Pennings. The c-Raf pS259 peptide was a kind gift from Dr Emira Visser. The ERRγ peptide was provided by Dr Bente Somsen. SSBP4, CIP2A, and RIPK2 peptides were provided by Yannick Leurs. Fusicoccin analogues were provided by Dr Peter Cossar and Dr Sebastian Andrei.[1,2] ERRγ reactive stabilizers were provided by Dr Peter Cossar and Siebe van der Elzen.[3] Full chemical identity of 14-3-3-binding peptides is given in Table S1. The PPARγ coregulators are shown in Table S2. EP300 was synthesized in house for which Justin Houx is thanked. PRIPRAP and MED1 DRIP2 were purchased from Thermo Scientific. Unless otherwise noted, all other reagents were obtained from commercial suppliers and used without further purification. ^{1}H NMR spectra were collected on an AVANCE III HD (400 MHz) NMR spectrometer (Bruker). The ^{1}H NMR chemical shift values are reported in ppm relative to the residual solvent peak.

DNA molecular biology and cloning

Protein sequences and physicochemical properties are given in Table S3. All DNA was ordered through Integrated DNA Technologies (IDT). The constructs were codon-optimized using IDT's built-in codon optimization tool for *Escherichia coli* (*E. coli*). The pET28a vector and gBlock dsDNA fragments were digested with the appropriate restriction enzymes (New England Biolabs). After ligation into the vector, the constructs were verified using Sanger sequencing (Azenta). Constructs were transformed into BL21(DE3) *E. Coli* cells (Novagen).

Expression of 14-3-3γ-SmBiT, LgBiT-ERRγ, FROG/B, and mCherryEA

For protein expression, 1 L or 0.5 L of 2xYT medium supplemented with 30 µg mL^{-1} of kanamycin was used. After inoculation using an overnight culture grown at 37 °C, 250 rpm, the culture was grown to an optical density (OD600) of 0.6 at 37 °C, 140 rpm. Then, protein expression was induced by addition of isopropyl β-D-1-thiogalactopyranoside (IPTG) at a final concentration of 0.5 mM, with incubation overnight at 20 °C, 140 rpm. Cells were harvested by centrifugation at 4°C and 10000x*g* for 15 minutes. The cell pellets were resuspended in lysis buffer (50 mM Tris, 300 mM NaCl, 30 mM imidazole, pH 8.0 supplemented with 1 µL/10 mL benzonase). Cells were lysed using an EmulsiFlexC3 High-Pressure homogenizer (Avestin) at 15,000 psi for three consecutive rounds. Cell debris and insoluble proteins were removed by centrifugation at 4 °C and 35000x*g* for 20 minutes. His-tagged proteins were purified from the soluble lysate using Ni-NTA affinity chromatography (His-Bind Resin, Novagen). The lysate was loaded onto the His-bind resin and washed twice with wash buffer (50 mM Tris, 300 mM NaCl, 60 mM imidazole, pH 8.0). The His-tagged proteins were eluted from the resin using elution buffer (50 mM Tris, 300 mM NaCl, 250 mM imidazole, pH 8.0). The eluted fractions were analyzed using SDS-PAGE (4–20% Mini-PROTEAN TGX Precast Protein Gel, Bio-Rad) and the purest fractions were pooled. The combined fractions were extensively dialyzed against condensate buffer (20 mM HEPES, 100 mM KCl, pH 7.5, freshly prepared with 100 µM tris(2-carboxyethyl)phosphine (TCEP) using membrane tubing with a molecular weight cut-off (MWCO) of 12-14 kDa (Fisher Scientific). Protein concentration was determined using an ND-1000 spectrophotometer (Thermo Scientific) at 280 nm with theoretical extinction coefficients as determined by the Expasy ProtParam tool as shown in Table S2. The purified proteins were aliquoted, flash-frozen in liquid N_2, and stored at -80°C for single-use aliquots. The identity and purity of the protein samples were confirmed using liquid chromatography quadrupole time of flight mass spectrometry (LC–MS Q-ToF) in Figures S28-S31.

Expression of PKA (catalytic subunit)

2 L of TB auto-induction medium supplemented with kanamycin (30 µg mL-1) was inoculated using an overnight culture grown at 37 °C, 250 rpm. The culture was grown at 37 °C, 140 rpm, for 4 hours, after which protein expression was carried out overnight at 25 °C, 140 rpm. Cells were harvested by centrifugation at 4°C and 10000 xg for 15 minutes. The cell pellet was resuspended in lysis buffer (50 mM Tris, pH 8, 600 mM NaCl, 30 mM imidazole, 5% glycerol, pH 8.0 supplemented with 1 µL/10 mL benzonase). Cells were lysed using an EmulsiFlexC3 High-Pressure homogenizer (Avestin) at 15,000 psi for three consecutive rounds. Cell debris was removed by centrifugation at 4 °C and 35000 xg for 20 minutes. PKA was purified from the soluble lysate using Ni-NTA affinity chromatography (His-Bind Resin, Novagen). The lysate was loaded onto the His-bind resin and washed twice with lysis buffer (50 mM Tris, pH 8, 600 mM NaCl, 30 mM imidazole, 5% glycerol, pH 8.0). The His-tagged proteins were eluted from the resin using elution buffer (50 mM Tris, pH 8, 600 mM NaCl, 250 mM imidazole, 5% glycerol, pH 8.0). Subsequently, the fractions were loaded on a pre-equilibrated 2 mL Strep-Tactin XT column (Iba Lifesciences). After 2 repeats of 5 column volumes of washing with wash buffer (100 mM Tris pH 8, 150 mM NaCl, 1 mM EDTA), the protein was eluted using freshly prepared Strep elution buffer (100 mM Tris pH 8, 150 mM NaCl, 1mM EDTA, 50 mM biotin). The eluted fractions were analyzed using SDS-PAGE (4–20% Mini-PROTEAN TGX Precast Protein Gel, Bio-Rad) and the purest fractions were pooled. The protein was extensively dialyzed against storage buffer (50 mM HEPES, 100 mM KCl, pH 7.5) using membrane tubing with a MWCO of 12-14 kDa (Fisher Scientific). Protein concentration was determined using an ND-1000 spectrophotometer (Thermo Scientific) at 280 nm with a theoretical extinction coefficient of 59270 M^{-1} cm^{-1} as determined by the Expasy ProtParam tool. The protein was aliquoted into single-use aliquots, flash-frozen in liquid N_2, and stored at -80°C. The identity and purity of the protein sample were confirmed using LC–MS Q-ToF (Figure S32).

Expression of PPARγ ligand binding domain

For the expression of PPARγ LBD, 3x 2 L of TB medium supplemented with ampicillin (30 µg/mL) was used. After inoculation using overnight cultures grown at 37 °C, 250 rpm, the culture was grown to an optical density (OD600) of 0.8-1.0 at 37 °C. Then, protein expression was induced by addition of isopropyl β-D-1-thiogalactopyranoside (IPTG) at a final concentration of 0.1 mM, with incubation overnight at 18 °C, 140 rpm. Cells were harvested by centrifugation at 4°C and 15000x*g* for 10 minutes. The cell pellets were resuspended in lysis buffer (50 mM Tris, 300 mM NaCl, 25 mM imidazole, 5 mM $MgCl_2$, pH 8.0 supplemented with 1 µL/10 mL benzonase). Cells were lysed using an EmulsiFlexC3 High-Pressure homogenizer (Avestin) at 15,000 psi for two consecutive rounds. Cell debris and insoluble proteins were removed by centrifugation at 4 °C and 40000x*g* for 35 minutes. His-tagged proteins were purified from the soluble lysate using Ni-NTA affinity chromatography (Ni-NTA 5 mL Superflow Cartridge, Qiagen). The lysate was loaded onto the column and washed for six column volumes with lysis buffer. The His-tagged proteins were eluted from the column using elution buffer (50 mM Tris, 300 mM NaCl, 200 mM imidazole, pH 8.0). For further purification, size exclusion chromatography (SEC) was carried out on a Bio-Rad NGC system connected to a HiLoad 16/600 Superdex 200 pg column at a flow rate of 1 mL min^{-1}, monitoring the absorbance at 280 nm. The column was equilibrated with SEC buffer (25 mM HEPES, 100 mM NaCl, 2 mM $MgCl_2$, 1 mM TCEP, pH 7.4 and 10 % w/v glycerol). The purest fractions were combined, and the protein was concentrated using an Amicon Ultra Centrifugal Filter (10 kDa MWCO, Millipore). Protein concentration was determined using an ND-1000 spectrophotometer (Thermo Scientific) at 280 nm with theoretical extinction coefficients as determined by the Expasy ProtParam tool as shown in Table S2. The purified proteins were aliquoted, flash-frozen in liquid N_2, and stored at -80°C for single-use aliquots. The identity

and purity of the protein were confirmed using liquid chromatography quadrupole time of flight mass spectrometry (LC–MS Q-ToF) in Figure S33.

Preparative phosphorylation of LgBiT-ERRγ

LgBiT-ERRγ was phosphorylated by PKA that was first treated with TEV protease (ProTEV Plus, Promega #V6101) to remove its His-tag according to the manufacturer's protocol. LgBiT-ERRγ, TEV-treated PKA and ATP were added at the molar ratio 1:0.02:10 in phosphorylation buffer (20 mM HEPES, 100 mM NaCl, 20 mM $MgCl_2$, pH 7.5) and incubated at 37°C for 50 min. After incubation, the solution was applied to a Ni-NTA gravity flow column. The flow-through containing TEV-cleaved PKA was discarded, and LgBiT-ERRγ pS was eluted, combined, and exchanged into condensate buffer (20 mM HEPES, 100 mM KCl, pH 7.5) by extensive dialysis using membrane tubing of 12-14 kDa (Spectra/Por® 2 RC Dialysis Membrane, Fisher Scientific). Protein concentration was determined using an ND-1000 spectrophotometer (Thermo Scientific) at 280 nm as determined by the online ProtParam tool (ExPASy). Aliquots of pure protein were flash-frozen and stored at -80°C. The degree of phosphorylation was analyzed using LC–MS Q-TOF (Figure S34)

LC-MS Q-ToF

Mass and purity of the proteins were determined using a high-resolution LC-MS Q-ToF system consisting of an ACQUITY UPLC I-Class system (Waters) coupled to a Xevo G2 quadrupole time of flight. The protein was separated (0.3 mL min^{-1}) on a column (Polaris C18A reverse phase column 2.0 × 100 mm, Agilent) using a 15–75% acetonitrile gradient in water supplemented with 0.1% v/v formic acid before analysis in positive mode in the mass spectrometer. The m/z spectra were deconvoluted using the MaxENTI algorithm in the Masslynx v4.1 software.

Labeling of proteins with fluorescent dyes

For aspecific fluorescent labeling of 14-3-3σ, 14-3-3γ, and PPARγ, *N*-hydroxysuccinimide (NHS) ester activated chemistry was used. DyLight 405 or AF 647 NHS ester (Lumiprobe) were dissolved at 10 mg/mL in DMSO. Proteins were diluted at least tenfold into labeling buffer (0.1 M $NaHCO_3$, pH 8.5) that was supplied with the dye directly before the protein was added. 1.5x to 3.0x of molar excess of dye relative to the protein was used, and the mixture was incubated at 4 °C for 3 hours. Unreacted dye was removed using twice a PD Minitrap G-25 size exclusion column, equilibrated with condensate buffer (20 mM HEPES, 100 mM KCl, pH 7.5). The average labeling per protein was measured using the absorption of the dye at their maximum absorption wavelength and at 280 nm for the protein, using the manufacturer-provided extinction coefficients and A_{280} correction factors for the dyes. The following average degrees of labeling were found: 1.8 in the case of 14-3-3σ labeled with AF 647, 0.6 in the case of 14-3-3σ labeled with DyLight 405, 1.7 in the case of 14-3-3γ labeled with AF 647, and 0.4 in the case of PPARγ labeled with AF 647.

Synthesis of amylose derivatives

Quaternized amylose (Q-Am), carboxymethylated amylose (Cm-Am), and nitrilotriacetic acid-modified amylose (NTA-Am) were synthesized using procedures based on those previously published.[4,5] The synthetic routes for amylose derivatives are shown in Scheme S1. For Q-Am, 12–16 kDa amylose (Carbosynth, 1.5 g) and NaOH (2.8 g) were dissolved in Milli-Q (14.25 mL) at 35 °C. After complete dissolution of the amylose, 3-chloro-2-

hydroxypropyltrimethylammonium chloride solution (11.64 mL, 60 wt% in water) was added dropwise into the stirring reaction mixture, which was subsequently stirred overnight at 35 °C. Next, the mixture was neutralized with acetic acid and precipitated into cold ethanol (200 mL). The resulting precipitate was re-dissolved in Milli-Q water and dialyzed extensively against water using regenerated cellulose dialysis tubing (Spectrum Labs, USA) with a 3.5 kDa MWCO before lyophilization. This yielded Q-Am as colorless solid (5 g, ca. 80 % yield), with a degree of substitution of 0.8 as determined by ^{1}H NMR (D_2O) in Figure S35. For the LC-MS experiment to determine FC partitioning, a separate Q-Am batch was used due to insufficient material of the main Q-Am batch. The same procedure was used, and the degree of substitution was similar (0.7), as determined by ^{1}H NMR (D_2O) in Figure S36.

In the case of Cm-Am, 12–16 kDa amylose (Carbosynth, 1.5 g) and NaOH (3.6 g) were dissolved in Milli-Q (15 mL) at 35 °C. After complete dissolution of the amylose, chloroacetic acid (2.7 g) was added dropwise into the stirring reaction mixture, which was subsequently stirred for 2 h at 35 °C. Next, the mixture was neutralized with acetic acid and precipitated into cold ethanol (200 mL). The resulting precipitate was re-dissolved in Milli-Q water and dialyzed extensively against water using regenerated cellulose dialysis tubing (Spectrum Labs, USA) with a 3.5 kDa MWCO before lyophilization. This yielded Cm-Am as colorless solid (5 g, ca. 80% yield), with a degree of substitution of 0.4 as determined by ^{1}H NMR (D_2O) in Figure S37.

Nitrilotriacetic acid-modified amylose (NTA-Am) was prepared via EDC/NHS activation of the Cm-Am carboxylic acid (Scheme S1), followed by amide bond formation with an amine-functionalized NTA. First, Cm-Am (85 mg, 0.39 mmol eq.) was dissolved in 10 mM $NaHPO_4$ buffer (10 mL) adjusted to pH 6 with 1 M HCl. To this was added *N*-hydroxysuccinimide (67 mg, 0.58 mmol) and 1-ethyl-3-(3-dimethylaminopropyl)carbodiimide (222 mg, 1.16 mmol). The reaction mixture was then stirred for 2 h at room temperature. This mixture was subsequently concentrated using 3 kDa MWCO spin filters, diluted with 10 mM $NaHPO_4$ buffer (adjusted to pH 8), and concentrated again to remove reagents and change the pH of the reaction medium for the next step. This centrifuge/dilution cycle was repeated a further two times. For the conjugation of the NTA group, *Nα,Nα*-bis(carboxymethyl)-L-lysine hydrate (152 mg, 0.58 mmol) was first dissolved in 10 mM $NaHPO_4$ buffer (18 mL) with 5% DMSO and adjusted to pH 8. To this solution was added the NHS-activated Cm-Am, and the reaction mixture was left to stir at room temperature overnight. The reaction mixture was concentrated, dialyzed extensively against MilliQ water, and lyophilized to yield NTA-Am (160 mg, ca. 90%) as colorless solid with a degree of substitution of NTA groups of 0.09 as determined by ^{1}H NMR (D_2O) in Figure S38. In the ^{1}H NMR spectrum, signals corresponding to common EDC/NHS side reactions were observed; signals for β-alanine formation by NHS ring opening and signals for N-acylurea groups were found.[6]

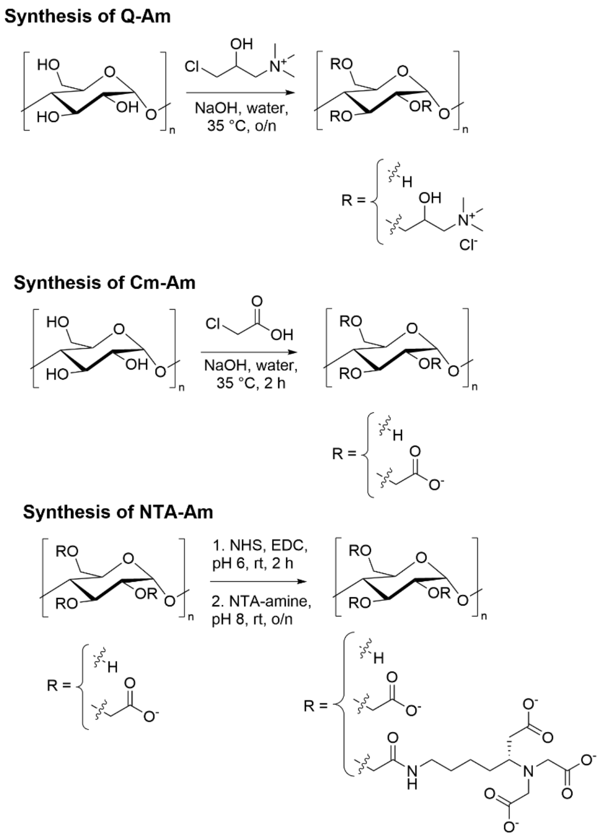

Scheme S1. Synthetic routes for amylose derivatives used in this work.

Synthesis of Cy5-labeled Cm-Am

Cy5-labeled Cm-Am was prepared via EDC/NHS activation of the Cm-Am carboxylic acid (Scheme S2), followed by amide bond formation with 3-azido-1-propanamine. First, Cm-Am (50 mg, 0.23 mmol eq.) was dissolved in 10 mM MES buffer (10 mL) at pH 6. To this was added *N*-hydroxysulfosuccinimide as a sodium salt (74 mg, 0.34 mmol) and 1-ethyl-3-(3-dimethylaminopropyl)carbodiimide (129 mg, 0.68 mmol). The reaction mixture was then stirred for 2 h at room temperature. This mixture was subsequently concentrated using 3 kDa MWCO spin filters, diluted with 10 mM $NaHPO_4$ buffer (adjusted to pH 8), and concentrated again to remove reagents and change the pH of the reaction medium for the next step. This centrifuge/dilution cycle was repeated a further two times. Next, 3-azido-1-propanamine (45 µL, 0.46 mmol) was added to the Cm-Am solution, and the pH was adjusted to pH 8. The reaction mixture was left to stir at room temperature overnight. The reaction mixture was concentrated, dialyzed extensively against MilliQ water, and lyophilized to yield N_3/Cm-Am (ca. 80% yield) as colorless solid. The amide coupling was confirmed to be successful as determined by ^{1}H NMR spectroscopy (D_2O) in Figure S39. In the ^{1}H NMR spectrum, signals corresponding to the alkyl groups of the azidopropanamide group were found at 1.8 and 3.4 ppm. Next, the N_3/Cm-Am (12 mg, 0.86 µmol estimated azide groups) was dissolved in 40 µL of MilliQ, to which was added 10.2 µL of a DMF solution of DBCO-Cy5 (10 mg/mL, 0.086 µmol). The reaction was stirred overnight at room temperature and dialyzed extensively against 20 v/v% DMF in MilliQ water and subsequently 100% MilliQ water, and lyophilized to yield Cy5-labeled Cm-Am as blue fluffy solid (5 mg, 42% yield) without further characterization.

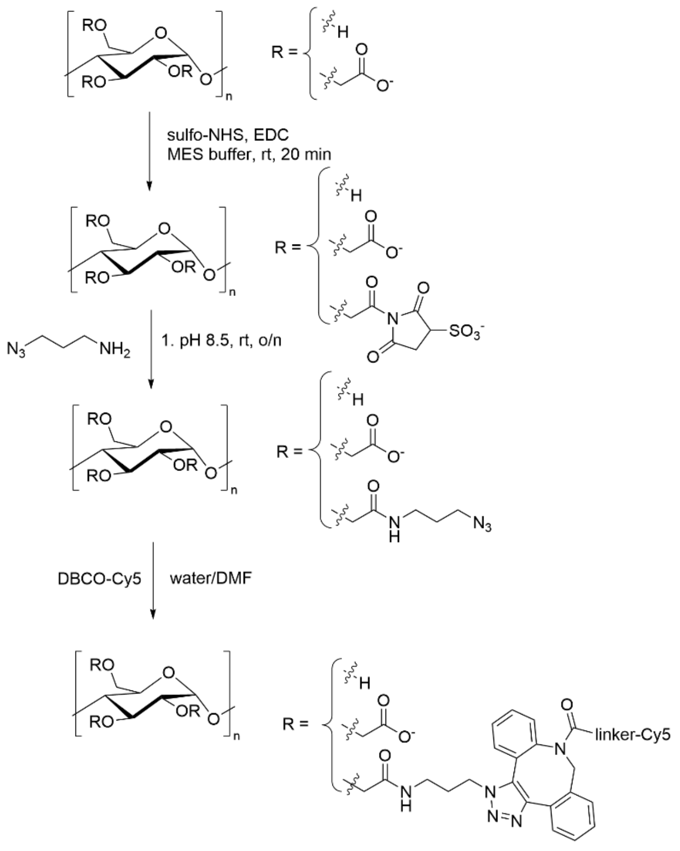

Scheme S2. Synthetic route for Cy5-labeled Cm-Am.

Synthesis of poly(ethylene glycol)-poly(caprolactone-gradient-trimethylene carbonate)-polyglutamic acid (PEG-b-PCLgPTMC-b-PGA) terpolymer

The terpolymer (PEG-b-PCLgPTMC-b-PGA) was synthesized as described by a previously published procedure.[4] Step 1: Preparation of poly(ethylene glycol)-poly(caprolactone-gradient-trimethylene carbonate) (PEG-PCLgTMC). The organocatalyzed ring-opening polymerization of ε-caprolactone and trimethylene carbonate was performed, aiming for a composition of PEG_{44}-PCL_{50}-g-TMC_{50}. Monomethoxy-PEG-OH macroinitiator (2 kDa, 0.5 mmol) was weighed into an oven-dried round bottom flask and dried at 140 ºC under vacuum. After cooling down, distilled ε-caprolactone (ε-CL, 25 mmol) and recrystallized trimethylene carbonate (TMC, 25 mmol) were added under argon and dissolved in dry DCM (12 mL). The reaction was initiated by the addition of methanesulfonic acid (0.25 mmol ≈ 125 μL). The reaction mixture was stirred at 25 °C in a water bath and reaction progress was regularly monitored by ^{1}H NMR spectroscopy. After completion (4-6 h) the reaction was quenched with DIPEA (1.5 mL), and the polymer was precipitated into ice cold methanol and lyophilized. This yielded 6.7 g of a waxy solid (67% yield). GPC analysis (using a PL gel 5 μm mixed D column, with THF as the solvent and calibration using polystyrene standards) yielded a polydispersity of 1.2.

Step 2: Chain-end modification with Boc-L-phenylalanine and deprotection. PEG-PCLgTMC (49 μmol) was dissolved in 5 mL acetonitrile. Then DMAP (25 μmol) and Boc-L-phenylalanine (0.25 mmol) were added, and the solution was cooled to 0 ºC. After that, DCC (1.2 mmol) dissolved in ACN (1 mL) was added dropwise to the mixture. The reaction was stirred for 24 h at RT. After reaction completion the flask was placed in the freezer for 1 h and filtered through a plug of celite. The filtrate was concentrated and precipitated in cold Et_2O (50 mL) to obtain the pure product. The resulting copolymer was then dissolved in DCM (5 mL), to which trifluoro acetic acid (5 mL) was added (on ice). The mixture was allowed to warm to RT and stirred for 2 h. After 2 h the solvent was evaporated and the copolymer was washed with $NaHCO_3$, 1 M NaCl and brine. Then it was dried over $MgSO_4$, filtered, concentrated, and finally precipitated from ice cold Et_2O. ^{1}H NMR spectroscopy showed that the signal arising from the terminal TMC group had disappeared, due to addition of phenylalanine at the terminus, and aromatic protons were visible at around 7.2-7.3 ppm. GPC analysis before and after deprotection yielded polydispersities of 1.1, indicating that TFA treatment did not facilitate copolymer hydrolysis.

Step 3: Polymerization and deprotection of N-carboxyanhydride γ-benzyl L-glutamate (BLG-NCA). Phe-terminated copolymer (57 μmol) was weighed into a Schlenk flask and dissolved in dry DMF (3 mL). Then NCA-BLG (5.7 mmol) was added under argon and the reaction was left under a constant flow of N_2 for 24 hrs. The product was precipitated into cold methanol and analyzed by ^{1}H NMR spectroscopy to confirm the overall composition and, in particular, the presence of benzylic and aromatic protons at 5.0-5.2 and 7.1-7.4 ppm, respectively. The resulting polymer (200 mg) was dissolved in THF (16 mL) and EtOH (2.5 mL) was added. The solution was degassed by bubbling N_2 through the solution for 20 min. Pd/C (10 mg) was added, the flask was filled with H_2 and the solution was left stirring overnight. After completion, the solution was filtered over celite. The filtrate was concentrated, precipitated into ice cold methanol, and lyophilized from dioxane. A colorless waxy solid was obtained. ^{1}H NMR spectroscopy was used to confirm successful deprotection of the PBLG units. The final composition of the terpolymer was $PEG_{44}P(CL_{63}gTMC_{52})PGA_{7}$. The composition of the resulting copolymer was confirmed by ^{1}H NMR spectroscopy (Figure S40), comparing the

protons of PEG (3.65 - 3.7 ppm), terminal methyl unit (singlet at 3.40 ppm) to PCL CH_2 (multiplet at 2.40 - 2.25 ppm) and PTMC CH_2 (multiplet at 2.2 - 1.8 ppm).

Condensate preparation

Condensates were prepared based on a previously reported procedure.[4] Q-Am, Cm-Am, and NTA-Am were dissolved separately in condensate buffer (20 mM HEPES, 100 mM KCl, pH 7.5) at a concentration of 1 mg mL^{-1}. First, buffer, BSA (final concentration 0.1 w/v%), and NTA-Am were added to 7.5 µM of $NiCl_2$ (final concentration) in a 1.5 mL tube, together with Cm-Am. The tube was placed in a MixMate shaker shaking at 1500 rpm (Eppendorf). Consecutively, Q-Am was added to induce coacervation in a 1.7:0.8:0.2 mass ratio of Q-Am:Cm-Am:NTA-Am, corresponding to a 2.5:0.8:0.2 charge ratio due to differing degrees of substitution, which was found to be the most stable. After 30 s, His-tagged protein cargo was added to the shaking solution. To achieve stabilized particles, 3.3 µL terpolymer (50 mg mL^{-1} in methoxy-poly(ethylene glycol) 350 on condensate volume of 100 µL) was added after 6 min and the mixture was shaken for another 5-10 s. For microscopy, 25-100 µL of each sample was loaded on an 18 well microscopy slide with glass bottom (Ibidi). In the case of experiments at pH values below 7 or above 8, HEPES was replaced by other buffering agents, specifically MES (pH 5-6.5), Tris (pH 8.5), or CHES (pH 9-10), with their concentration kept at 20 mM.

LC-MS assay for partitioning of small molecules

Condensates were prepared using an adapted version of the normal protocol, since a highly concentrated and large condensate sample (1.1 mL) was needed for forming a condensate macrophase of sufficient scale (>10 µL). For this experiment, a different but similar batch of Q-Am was used because of the large amount of material needed (see synthetic procedure for Q-Am). Q-Am and Cm-Am were dissolved separately in condensate buffer (20 mM HEPES, 100 mM KCl, pH 7.5) at a concentration of 10 mg mL^{-1}. NTA-Am was omitted since it was not available in a large quantity to perform the experiment. First, buffer, BSA (final concentration 0.1 w/v%), and Cm-Am were added to a 1.5 mL tube. The tube was placed in a MixMate shaker shaking at 1500 rpm (Eppendorf). Consecutively, Q-Am was added to induce coacervation in a 1.7:1 mass ratio of Q-Am:Cm-Am, corresponding to a 2.5:1 charge ratio due to differing degrees of substitution. The solution was shaken for 6 minutes, after which it was removed from the shaker and 100 µM of FC was added. The sample was analyzed on a brightfield microscope to confirm the presence of condensates. The sample was incubated for 2 h to allow for equilibration of FC. Next, the sample was centrifuged to form a condensate macrophase (25 °C, 21300xg, 10 min). The supernatant was carefully removed from the condensate macrophase, consisting of ~15 µL. Next, a liquid-liquid extraction step was carried out to selectively extract FC without proteins and polymers. 10 µL of both the supernatant and the condensate macrophase were separately diluted with 90 µL of 1 M NaCl solution, to dissolve the condensate macrophase, in a 1.5 mL tube. Next, the aqueous solution was extracted with 250 µL of dichloromethane by means of vortexing for 30 s. The dichloromethane layer was carefully isolated and evaporated under a stream of argon. Next, the residue, containing FC, was dissolved in 100 µL of MilliQ/acetonitrile 95/5 v/v% supplemented with 0.1% formic acid (FA) for LC-MS. LC-MS analysis was carried out with a system comprising a Phenomex kinetex® 2.6 µm EVO C18 50 x 2.1 mm column using a mixture of ultrapure water with 0.1% FA and acetonitrile with 0.1% FA. For this experiment, a custom method with isocratic elution at 35%

acetonitrile was used. The system was connected to a Thermo Fisher LCQ Fleet Ion Trap Mass Spectrometer. 4 µL of the samples was injected, and the runs were performed for a total of three technical replicates. The data was processed as shown in Figure S9, after which the relative FC signal was used to determine the partitioning coefficient.

Fluorescence anisotropy (FA) assays

14-3-3 or PPARγ were titrated in a 2-fold dilution series to 10 of 100 nM of fluorescently labeled peptides. For 14-3-3, condensate buffer was used (20 mM HEPES, 100 mM KCl, pH 7.5) supplemented with 0.1% (v/v) of Tween 20 and 1 mg/mL of bovine serum albumin (BSA) to prevent aspecific hydrophobic interactions. For PPARγ the same buffer was used with 100 mM of NaCl instead of 100 mM of KCl. Dilution series were prepared in low volume, non-binding polystyrene 384 well plates (Corning 4514 Black Round Bottom 384-well plates). Measurements were performed directly after plate preparation using a Tecan Spark plate reader at room temperature. The following settings were used for the FITC peptides: excitation 485 ± 20 nm; emission: 535 ± 25 nm; mirror: Dichroic 510; number of flashes: 30; integration time: 40 µs; settle time: 1 ms; gain: optimal; and Z-position: calculated from well. For RBITC-labeled HSPB6, the following settings were used: excitation 535 ± 25 nm; emission: 590 ± 20 nm; mirror: Dichroic 560; number of flashes: 30; integration time: 40 µs; settle time: 1 ms; gain: optimal; and Z-position: calculated from well. Wells containing only the labelled peptide were used to set as G-factor at 35 mAU. All data were analyzed using GraphPad Prism (version 10.0.3) and fitted using a four-parameter logistic model (4PL) to determine binding affinities (K_D). All results are based on triplicates, with the mean and standard error determined by GraphPad.

Brightfield microscopy

Brightfield images were acquired using a Zeiss Axio Observer D1 microscope coupled with an AxioCamMR3 camera, at an objective with 20× magnification.

Confocal laser scanning microscopy

Confocal laser scanning microscopy (Leica TCS SP8) was used for analysis of condensates with fluorescent cargo. The system was equipped with a 405 nm laser (used for FROG/B), a 488 nm laser (used for FITC and mCherryEA), 552 nm laser (used for TAMRA, SNARF-4F, and mCherryEA), and 638 nm laser (used for AF 647) and a hybrid detector (HyD). For the 488 nm laser channel, emission was collected between 498 and 550 nm. For the 552 laser channel, emission was collected between 562 and 630 nm. Finally, for the 638 laser channel, emission was collected between 648 and 710 nm. Laser power and detector gain were optimized for each different construct and concentration to use the maximum number of gray values of the detector. For single timepoint measurements, an HC PL APO CS2 63× water immersion objective with a numerical aperture (NA) of 1.20 was used. Images were acquired with a resolution of 1024 × 1024 pixels and a pixel dwell time of 1.2 µs. For kinetic measurements, an HC PL APO CS2 20x dry objective with an NA of 0.75 was used. Images were acquired with a resolution of 1024 × 1024 or 512 × 512 and a scanning speed of 100 Hz at specified timepoints on certain positions using the Mark and Find tool. The pinhole was set to 1 Airy Unit for the wavelength of maximum emission for each fluorophore.

Image processing and analysis

All images were processed and analyzed with Fiji (ImageJ). For micrographs of the 14-3-3 and PPARγ channels, the brightness was digitally adjusted equally for enhanced visibility. The channels of the client were not adjusted and uncropped images in the SI were not adjusted either. For quantification of the internal fluorescence intensity, a threshold was applied to images in the 14-3-3 channel, converting it into a binary image. Next, the images were dilated using a maximum filter of radius 1 pixel, to make particle outlines more pronounced and particle recognition more reliable. Next, a watershed function was applied to separate adjacent condensates into individual regions of interest (ROIs). Using the particle analysis tool with appropriate cutoff values to select condensates as ROIs, fluorescence intensity was quantified. The ROIs recognized in the 14-3-3 channel were also redirected to the client peptide or protein channels, which is especially important for experiments over time since the 14-3-3 channel is relatively constant in fluorescence. Alternatively, for images without 14-3-3, Cy5-labeled Cm-Am was used to determine the condensate outlines. ROIs were visually checked to make sure that only condensates were selected. Next, recognized ROIs were filled and measured using the particle analysis tool, redirected to the peptide channel. The intensity was determined for each selected particle. For extensive procedures, the reader is referred to earlier work.[7]

Crystallography figures

Figures of crystal structures were generated using Chimera 1.17.1.[8]

Table S1. Overview of 14-3-3-binding peptides used in this work. Ahx = aminohexanoic acid, O1pen = (2-aminoethoxy)acetic acid, p = phosphate. FC = Fusicoccin A.

Peptide name	Amino acid sequence with modifications	Binding mode	Molecular glue sensitivity
c-Raf pS259	FITC-Ahx-QRSTpSTPNVH-Ac	Groove-filling	Not sensitive to FC
CIP2A	FITC-Ahx-KINPETVNLpSI-COOH	C-terminal	FC
ERRγ	FITC-Ahx-KRRRKpSCQA-Ac	C-terminal	Aldehyde/disulfide
HSPB6	RBITC-O1Pen-WLRRApSAPLPGLSAP-Ac	Groove-filling	Not sensitive to FC
RIP2K	FITC-Ahx-PSLNLLQNKpSM-COOH	C-terminal	FC
SSBP4	FITC-Ahx-ESYSPGMTMpSV-COOH	C-terminal	FC

Table S2. Overview of PPARγ-binding peptides used in this work. βA = β-alanine.

Peptide name	Amino acid sequence	Notes
EP300	FITC-βA-AASKHKQLSELLRSGSSPN-NH_2	Synthesized in house
MED1 DRIP-2	Fluorescein-NTKNHPMLMNLLKDNPAQD	Commercial product, PV4549, lot nr: 2307248B
PRIPRAP	Fluorescein-VTLTSPLLVNLLQSDISAG	Commercial product, PV4604, lot nr: 2081640E

Table S3. Amino acid sequences and physicochemical parameters of the proteins used in this work. Strep-tags are shown in blue, His-tags are shown in red, TEV protease sites are shown in orange, and 14-3-3-binding domains are shown in green. Text in **bold** shows thrombin cleavage sites. Underlined residues indicate residues that can be phosphorylated. Physicochemical parameters were calculated using the online ProtParam tool (ExPASy). The extinction coefficient (ε) is given with all Cys residues reduced. [a)]Mass without phosphorylated residues.

Protein name	Amino acid sequence	Theoretical PI	M_w (kDa)	ε (M^{-1} cm^{-1}) at 280 nm
14-3-3σ	SYYHHHHHHDYDIPTTENLYFQGAMGSMERASLIQKAK<br>LAEQAERYEDMAAFMKGAVEKGEELSCEERNLLSVAY<br>KNVVGGQRAAWRVLSSIEQKSNEEGSEEKGPEVREYRE<br>KVETELQGVCDTVLGLLDSHLIKEAGDAESRVFYLKMK<br>GDYYRYLAEVATGDDKKRIIDSARSAYQEAMDISKKEM<br>PPTNPIRLGLALNFSVFHYEIANSPEEAISLAKTTFDEAM<br>ADLHTLSEDSYKDSTLIMQLLRDNLTLWTADNAGEEGG<br>EAPQEPQS	4.82	31.0	31860
14-3-3γ	SYYHHHHHHDYDIPTTENLYFQGAMGSMVDREQLVQK<br>ARLAEQAERYDDMAAAMKNVTELNEPLSNEERNLLSV<br>AYKNVVGARRSSWRVISSIEQKTSADGNEKKIEMVRAY<br>REKIEKELEAVCQDVLSLLDNYLIKNCSETQYESKVFYL<br>KMKGDYYRYLAEVATGEKRATVVESSEKAYSEAHEISK<br>EHMQPTHPIRLGLALNYSVFYYEIQNAPEQACHLAKTAF<br>DDAIAELDTLNEDSYKDSTLIMQLLRDNLTLWTSDQQD<br>DDGGEGNN	4.96	31.5	37820
14-3-3γ-SmBiT	MGHHHHHHDYDIPGTENLYFQGAMGSVDREQLVQKAR<br>LAEQAERYDDMAAAMKNVTELNEPLSNEERNLLSVAY<br>KNVVGARRSSWRVISSIEQKTSADGNEKKIEMVRAYRE<br>KIEKELEAVCQDVLSLLDNYLIKNCSETQYESKVFYLKM<br>KGDYYRYLAEVATGEKRATVVESSEKAYSEAHEISKEH<br>MQPTHPIRLGLALNYSVFYYEIQNAPEQACHLAKTAFDD<br>AIAELDTLNEDSYKDSTLIMQLLRDNLTLWTSDQQGTG<br>GNGSSGGVTGYRLFEEILGGSGGSWSHPQFEKGGS	5.21	34.0	41830
LgBiT-ERRγ	MGSSHHHHHHENLYFQGSSGVFTLEDFVGDWEQTAAY<br>NLDQVLEQGGVSSLLQNLAVSVTPIQRIVRSGENALKIDI<br>HVIIPYEGLSADQMAQIEEVFKVVYPVDDHHFKVILPYG<br>TLVIDGVTPNMLNYFGRPYEGIAVFDGKKITVTGTLWN<br>GNKIIDERLITPDGSMLFRVTINSGGSGGSGGSGGSGGSG<br>GSGGSGGSGGSGGTKRRRKSCQAGGSGGTGGSWSHPQF<br>EK	6.03	24.7	26390

PKA (catalytic subunit)	MGHHHHHHSSGENLYFQGSVKEFLAKAKEDFLKKWESPAQNTAHLDQFERIKTLGTGSFGRVMLVKHKETGNHYAMKILDKQKVVKLKQIEHTLNEKRILQAVNFPFLVKLEFSFKDNSNLYMVMEYVPGGEMFSHLRRIGRFSEPHARFYAAQIVLTFEYLHSLDLIYRDLKPENLLIDQQGYIQVTDFGFAKRVKGRTWTLCGTPEYLAPEIILSKGYNKAVDWWALGVLIYEMAAGYPPFFADQPIQIYEKIVSGKVRFPSHFSSDLKDLLRNLLQVDLTKRFGNLKNGVNDIKNHKWFATTDWIAIYQRKVEAPFIPKFKGPGDTSNFDDYEEEEIRVSINEKCGKEFSEFSSGSSGWSHPQFEK	8.71	42.8[a)]	60850
FROG/B	MGSKGEELFTGVVPILVELDGDVNGHRFSVRGEGEGDATNGKLTLKFICTTGKLPVPWPTLVTTLSYGVACFSRYPDHMKQHDFFKSAMPEGYVQERTIFFKDDGTYKTRAEVKFEGDTLVNRIELKGIDFKEDGNILGHKLEYNYNSHCDYITADKQKNGIKANFKIRHNVEDGGVQLADHYQQNTPIGDGPVLLPDNHYLCVQVKLSKDPNEKRDHMVLLEFRTAAGITLGMDELYKGGSENLYFQGSHHHHHH	6.09	28.9	21890
mCherryEA	MGHHHHHHDYDIPGTENLYFQGAMVSKGEEDNMAIIKEFMRFKVHMEGSVNGHEFEIEGEGEGRPYEGTQTAKLKVTKGGPLPFAWDILSPQFMYGSKAYVKHPADIPDYLKLSFPEGFKWERVMNFEDGGVVTVTQDSSLQDGEFIYKVKLRGTNFPSDGPVMQKKTMGWEASSERMYPEDGALKGEEKARLKLKDGGHYDAEVKTTYKAKKPVQLPGAYNVNIKLDITSHNEDYTIVEQYERAEGRHSTGGMDELYK	5.66	29.4	37360
PPARγ (ligand binding domain)	MGSSHHHHHHSSG**LVPRGS**HMESADLRALAKHLYDSYIKSFPLTKAKARAILTGKTTDKSPFVIYDMNSLMMGEDKIKFKHITPLQEQSKEVAIRIFQGCQFRSVEAVQEITEYAKSIPGFVNLDLNDQVTLLKYGVHEIIYTMLASLMNKDGVLISEGQGFMTREFLKSLRKPFGDFMEPKFEFAVKFNALELDDSDLAIFIAVIILSGDRPGLLNVKPIEDIQDNLLQALELQLKLNHPESSQLFAKLLQKMTDLRQIVTEHVQLLQVIKKTETDMSLHPLLQEIYKDLY	6.37	33.3	11920

Supplementary Data

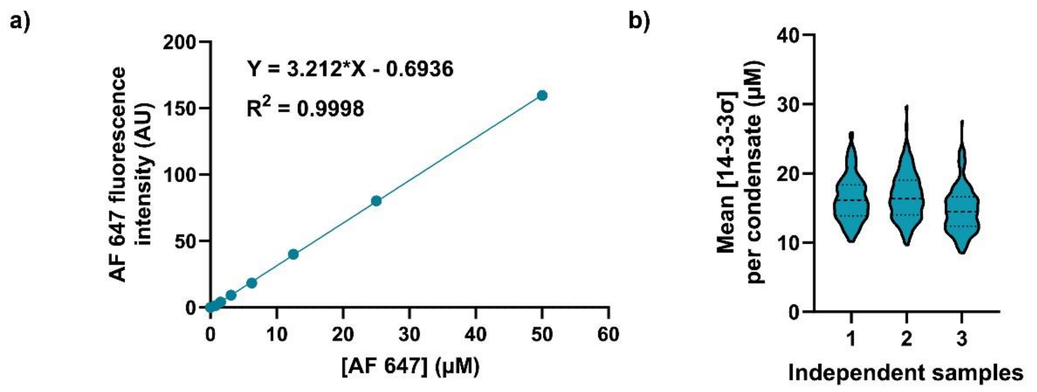

Figure S1. Quantification of the concentration of 14-3-3σ inside the condensates. a) Calibration curve of AF 647 in solution measured using the same settings as 14-3-3. The formula was obtained by linear regression. b) Quantification of 14-3-3σ (AF 647-labeled) across three independent condensate samples loaded with 100 nM of 14-3-3σ. In the calculation, the mean degree of labeling of 14-3-3σ with AF 647 was used, which was determined to be 1.69. The dashed lines show the mean, and the dotted lines show the quartiles. N ≥ 124 condensates.

Table S4. Estimation of the 14-3-3 concentration in several tissues. The mass fraction of 14-3-3 across tissues was determined by Boston et al.[9] We then used the estimated cellular protein concentration of 200 mg mL^{-1} to convert this into mg mL^{-1}.[10] Finally, the molar mass of human 14-3-3σ (27774 Da, UniProt ID P31947) was used to calculate the 14-3-3 concentration in µM.

Tissue	[14-3-3] (µg mg^{-1} total protein)[9]	[14-3-3] (mg mL^{-1})	[14-3-3] (µM)
Brain	13.3	2.66	95.8
Testis	6.0	1.2	43
Small intestine	5.9	1.18	42
Large intestine	2.8	0.56	20
Spleen	2.5	0.50	18
Adrenal	2.4	0.48	17
Prostate	2.2	0.44	16
Lung	1.0	0.20	7.2
Uterus	0.94	0.19	6.8
Liver	0.70	0.14	5.0
Heart	0.53	0.11	3.8
Pancreas	0.47	0.094	3.4
Skeletal muscle	0.28	0.056	2.0
Thyroid	0.12	0.024	0.86
Kidney	0.06	0.012	0.43

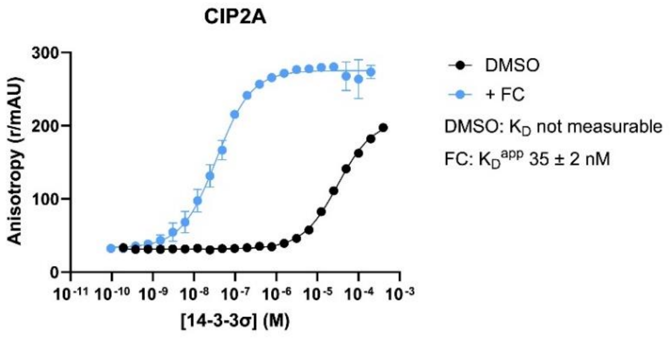

Figure S2. Protein titration of 14-3-3σ to the CIP2A peptide (10 nM), as measured by fluorescence anisotropy assay in the presence of DMSO (control) or 100 µM of FC. Symbols represent the mean of a technical triplicate, with the error bars partly obscured by the symbols. Lines show fits by a 4-parameter logistic model. The resulting K_D or K_D^{app} obtained from the fit is shown as mean with the standard error. For the DMSO sample, no reliable K_D could be fitted due to the absence of an upper plateau.

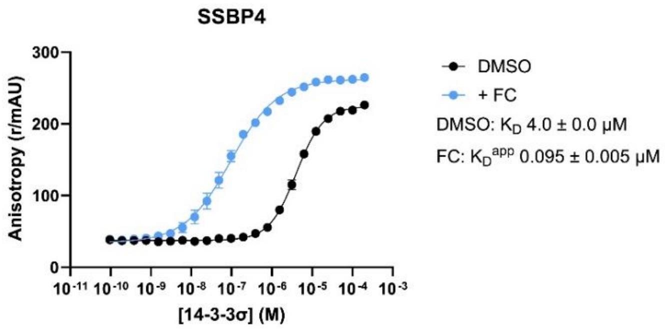

Figure S3. Protein titration of 14-3-3σ to the SSBP4 peptide (10 nM), as measured by fluorescence anisotropy assay in the presence of DMSO (control) or 100 µM of FC. Symbols represent the mean of a technical triplicate, with the error bars partly obscured by the symbols. Lines show fits by a 4-parameter logistic model. The resulting K_D or K_D^{app} obtained from the fit is shown as mean with the standard error.

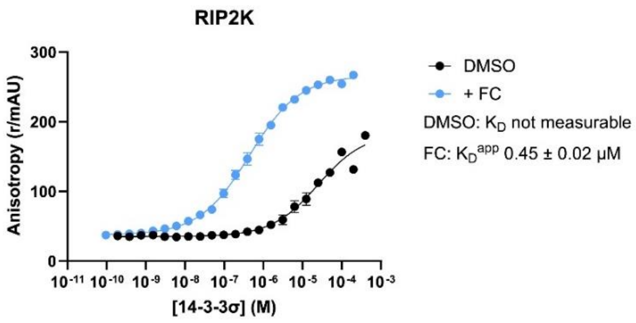

Figure S4. Protein titration of 14-3-3σ to the RIP2K peptide (10 nM), as measured by fluorescence anisotropy assay in the presence of DMSO (control) or 100 µM of FC. Symbols represent the mean of a technical triplicate, with the error bars partly obscured by the symbols. Lines show fits by a 4-parameter logistic model. The resulting K_D or K_D^{app} obtained from the fit is shown as mean with the standard error. For the DMSO sample, no reliable K_D could be fitted due to the absence of an upper plateau.

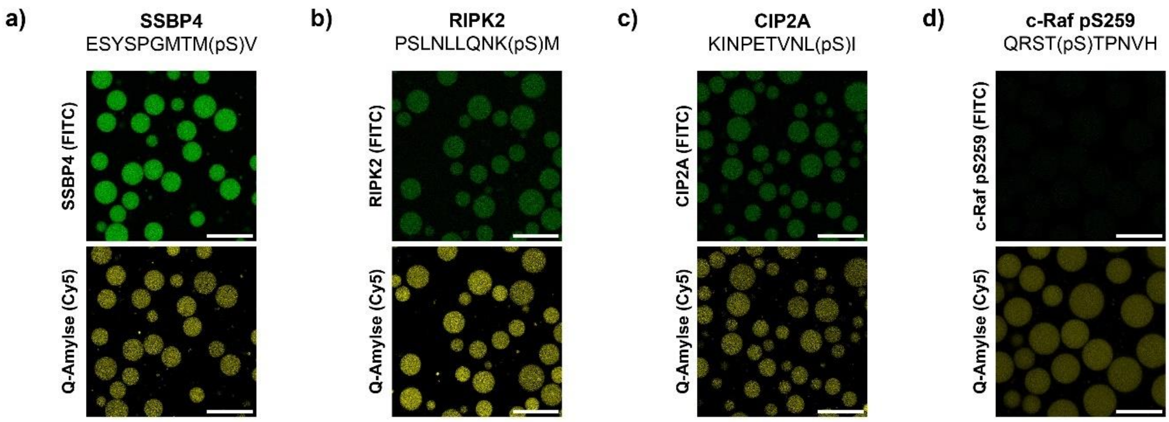

Figure S5. a-d) Confocal micrographs of condensates supplied with 100 nM of the peptides a) SSPB4, b) RIPK2, c) CIP2A, or d) c-Raf pS259. The condensates were prepared with Cy5-labeled Q-amylose to visualize them.

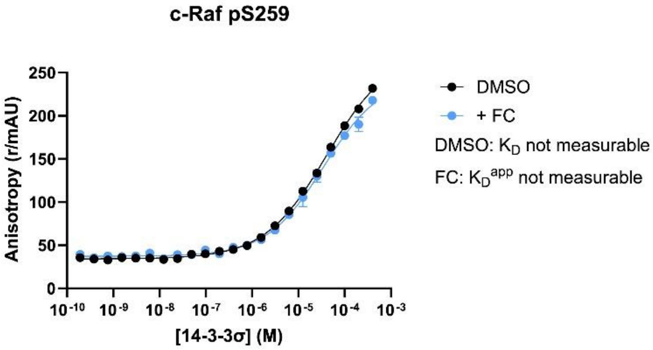

Figure S6. Protein titration of 14-3-3σ to the non-FC-responsive c-Raf pS259 peptide (10 nM), as measured by fluorescence anisotropy assay in the presence of DMSO (control) or 100 µM of FC. Symbols represent the mean of a technical triplicate, with the error bars partly obscured by the symbols. Lines show fits by a 4-parameter logistic model. For both titrations, no reliable K_D or K_D^{app} could be fitted due to the absence of an upper plateau.

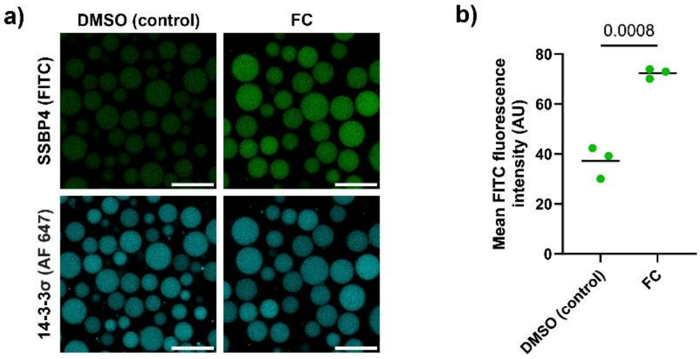

Figure S7. a) Confocal micrographs of 14-3-3σ-loaded condensates (10 nM of 14-3-3σ) supplied with SSBP4 (10 nM) in the absence (DMSO control) or presence of 100 µM of FC. Scale bar: 25 µm. b) Quantification of micrographs of samples used in panel a. Statistical analysis was performed by unpaired two-tailed t-test, with N ≥ 114 condensates across 2 imaging positions in 3 independent samples. P values are shown above the comparison. The line shows the mean, and the symbols show the individual measurements.

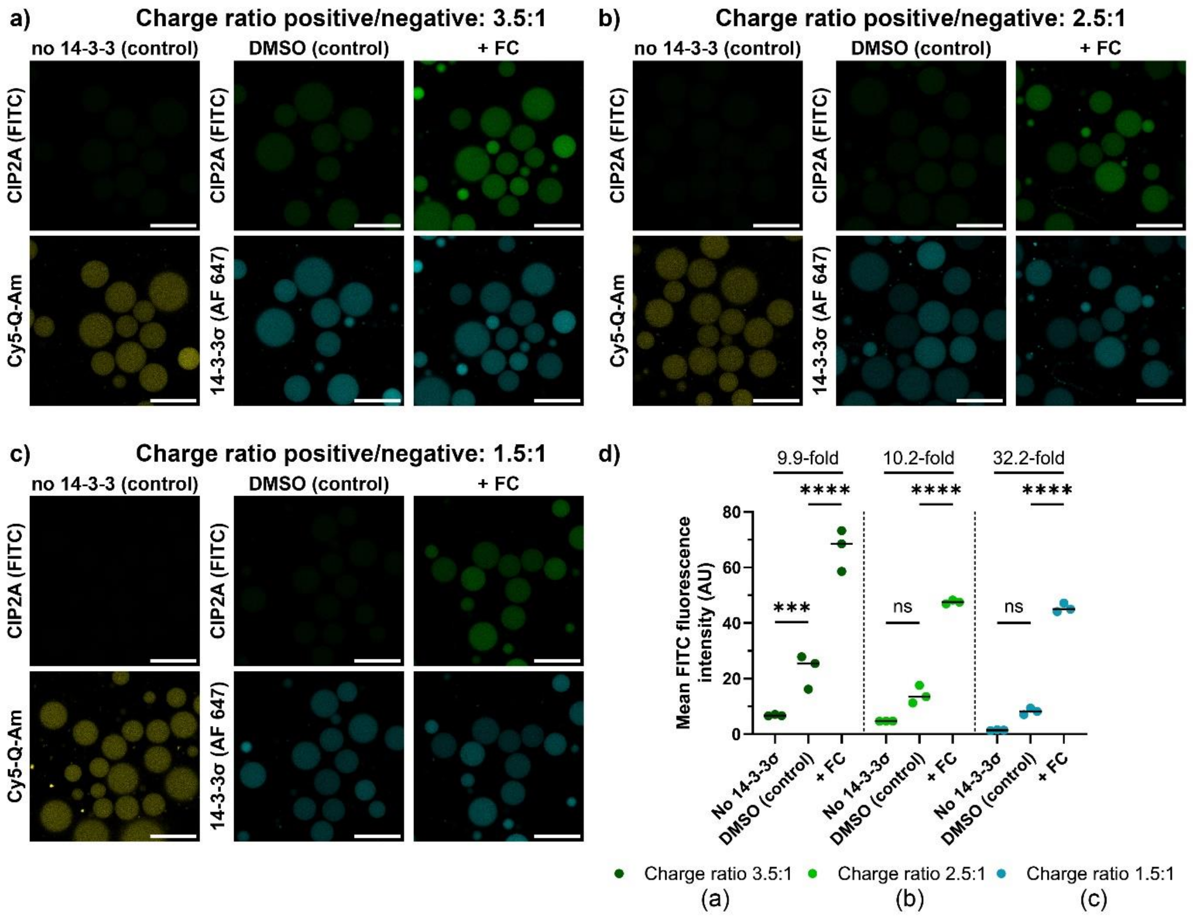

Figure S8. a-c) Confocal micrographs of 14-3-3σ-loaded condensates supplied with the CIP2A peptide (100 nM) in the absence (DMSO control) or presence of 100 µM of FC at various charge ratios between the polycationic and polyanionic amyloses. The samples were incubated overnight. Scale bar: 25 µm. d) Quantification of CIP2A samples in panels a-c. Statistical analysis was performed by one-way ANOVA with Tukey's test with correction for multiple comparisons, with $N \geq 44$ condensates across 2 imaging positions in 3 independent samples. P values are shown above the com-parison, with significance regarded as $P < 0.05$. The line shows the mean, and the symbols show the individual measurements. The fold change listed is the fold change in the mean.

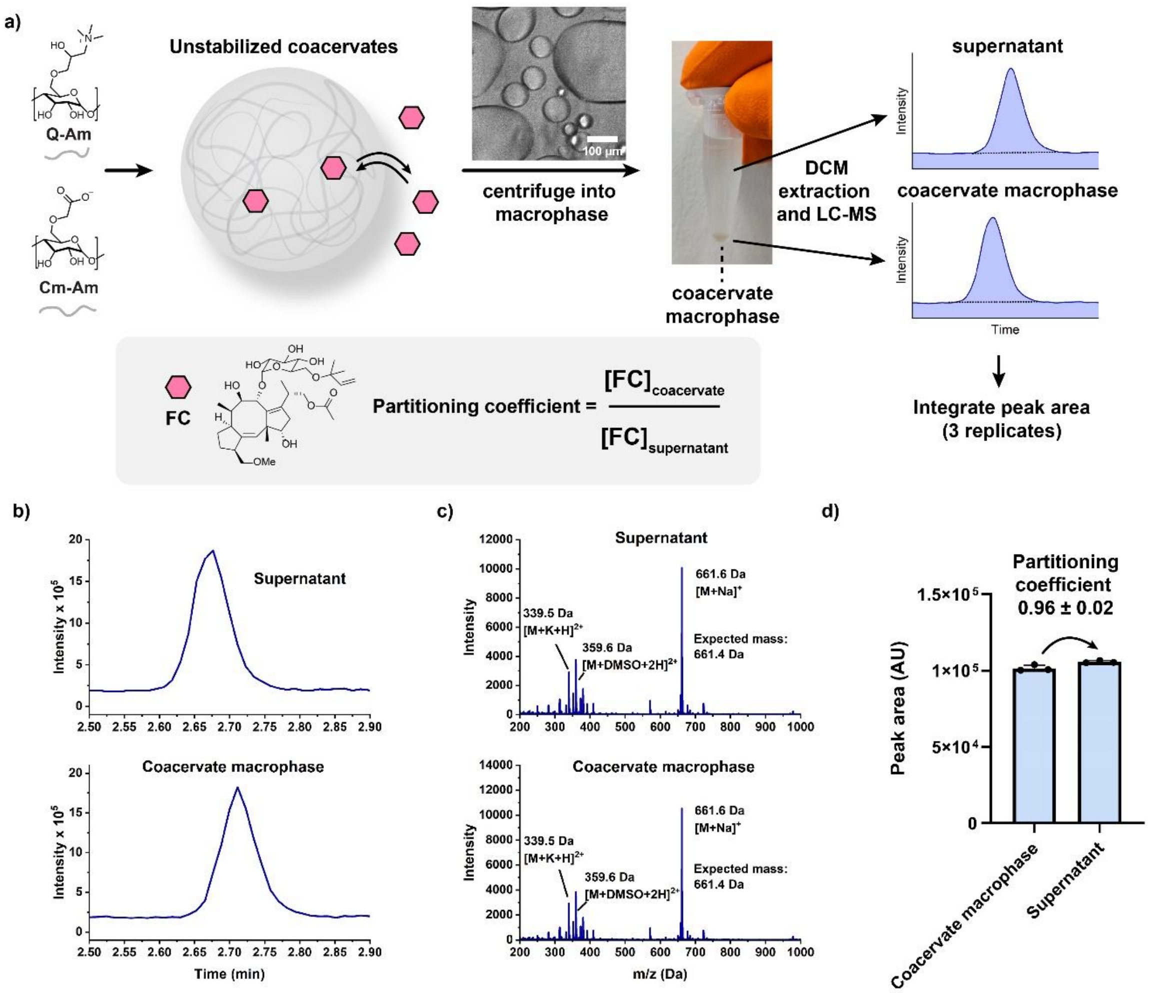

Figure S9. LC-MS assay for the determination of the partitioning coefficient of FC. a) Schematic overview of sample preparation and measurement of the partitioning coefficient. Condensates are prepared from Q-Am and Cm-Am at 10x the usual amylose concentration, yielding unstabilized condensates. The terpolymer was omitted because it is challenging to purify it for the LC-MS method, and the samples were pelleted into a condensate macrophase anyway. Ni-NTA-amylose was omitted due to the large scale of the experiment and insufficient material. A representative microscopy image taken directly after preparation is shown as inset, scale bar 100 µm. The sample was equilibrated and centrifuged to yield a condensate macrophase (photo of the sample shown), after which the relative concentration of FC in the supernatant and the condensate macrophase was determined by LC-MS. b) Chromatograms of the supernatant and condensate macrophase, after extraction, showing the FC peak. c) Mass spectra derived from the chromatograms shown in panel b. d) Determination of the partitioning coefficient by relative peak areas of the LC-MS chromatograms. N=3 technical replicates based on the same sample.

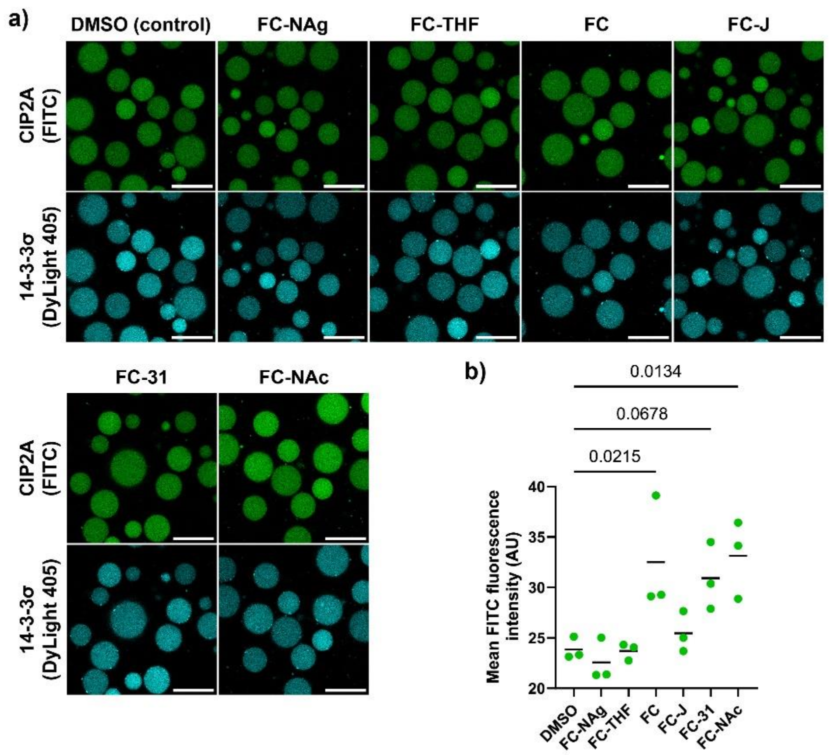

Figure S10. a) Confocal micrographs of 14-3-3σ-loaded condensates supplied with CIP2A (100 nM) in the absence (DMSO control) or presence of 1 µM of FC analogues. Scale bar: 25 µm. d) Quantification of micrographs of 14-3-3σ-loaded condensates and CIP2A (100 nM) in the absence (DMSO control) or presence of 1 µM of FC analogues. Statistical analysis was performed by one-way ANOVA with Dunnett's test with correction for multiple comparisons to the DMSO control, with N ≥ 48 condensates across 2 imaging positions in 3 independent samples. P values are shown above the comparison. The line shows the mean, and the symbols show the individual measurements.

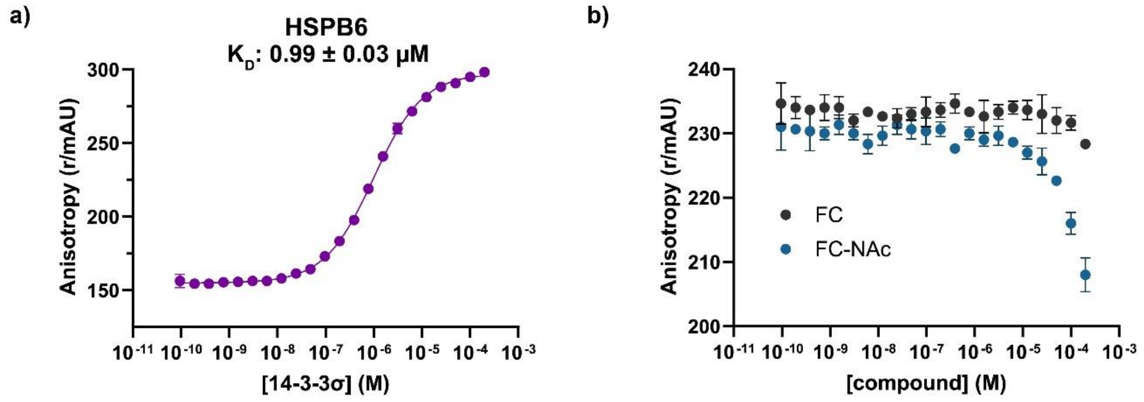

Figure S11. a) Protein titration of 14-3-3σ to the RBITC-labeled HSPB6 peptide (100 nM), as measured by fluorescence anisotropy assay. Symbols represent the mean of a technical triplicate, with the error bars partly obscured by the symbols. Lines show fits by a 4-parameter logistic model. The resulting K_D obtained from the fit, shown as mean with the standard error. b) Compound titration of FC or FC-NAc to RBITC-labeled HSPB6 (100 nM) in the presence of 14-3-3σ (1 µM). The highest concentration of compound is 200 µM in both cases. Symbols represent the mean of a triplicate measurement, with the error bars partly obscured by the symbols.

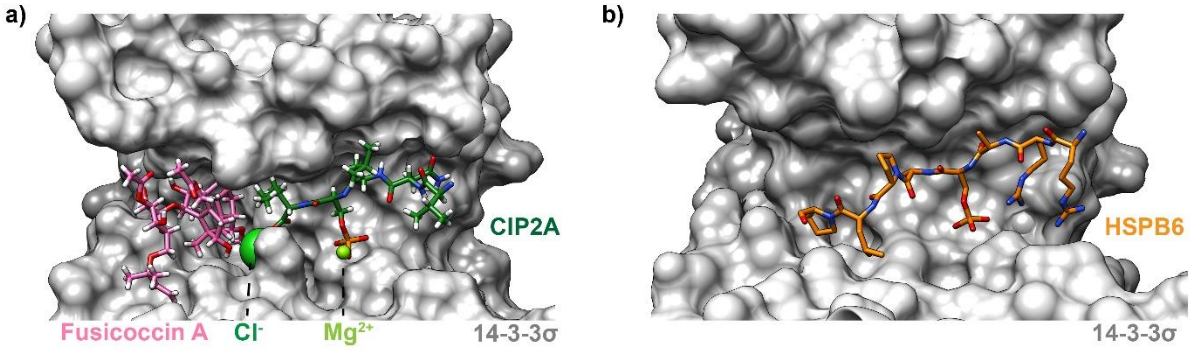

Figure S12. a) Crystal structure of 14-3-3σ/CIP2A interaction in the presence of Fusicoccin A. PDB: 7BMC.[11] b) Crystal structure of 14-3-3σ/HSPB6 interaction. The peptide extends into the Fusicoccin binding pocket. PDB: 5LU2.[12]

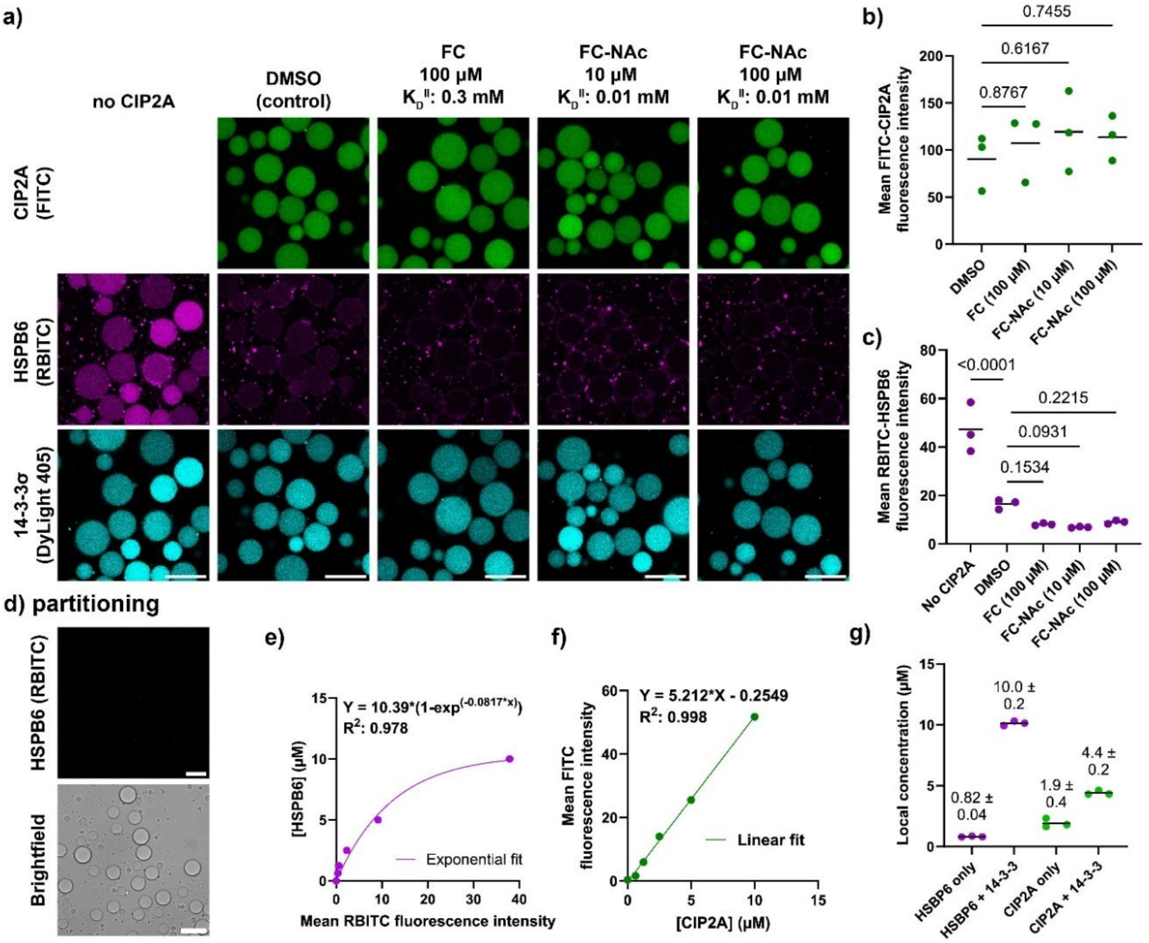

Figure S13. a) Confocal micrographs of 14-3-3σ-loaded condensates supplied with CIP2A and HSPB6 (150 nM each) in the absence (DMSO control) or presence of FC analogues. Scale bar: 25 μm. b, c) Quantification of b) CIP2A and c) HSPB6 in samples at the conditions of panel a. Statistical analysis was performed by one-way ANOVA with Tukey's test with correction for multiple comparisons, with N ≥ 53 condensates across 2 imaging positions in 3 independent samples. P values are shown above the comparison. The line shows the mean, and the symbols show the individual measurements. d) Condensates with HSPB6 peptide only (100 nM). e,f) Calibration curve of fluorescent peptides HSPB6 (e) and CIP2A (f) in solution. g) Calculation of local concentrations of peptides using data from Figure 2 (CIP2A) and this figure (HSPB6). The mean local concentration across 3 independent condensate samples is shown with the standard deviation. The line shows the mean.

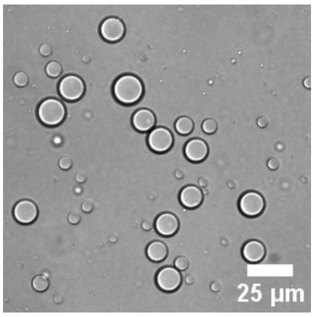

Figure S14. Brightfield micrograph of a representative condensate sample for the split luciferase ERRγ assay in condensates.

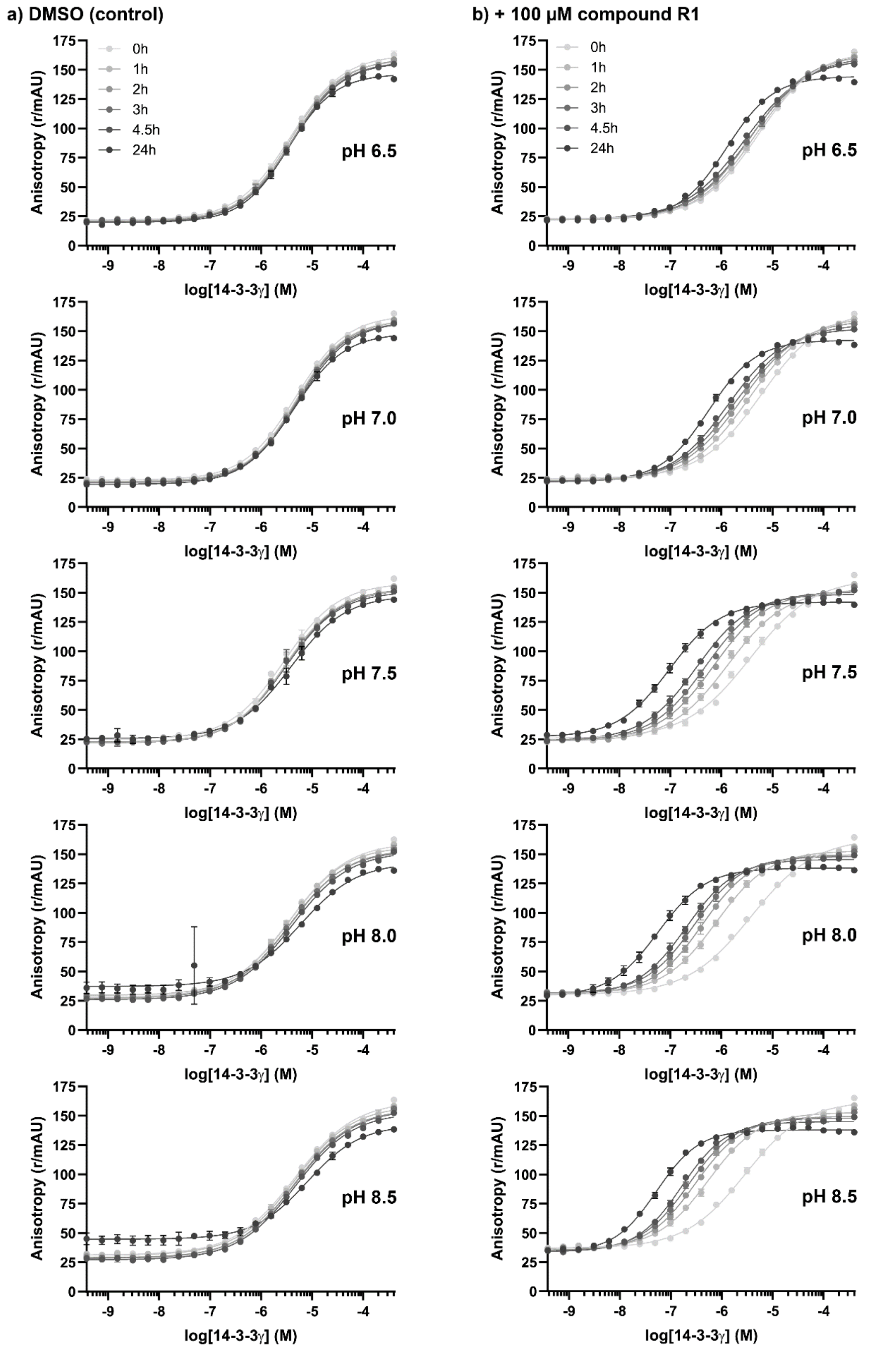

Figure S15. Time-dependent and pH-dependent protein titration of 14-3-3γ to 100 nM of FAM-labeled ERRγ peptide, as measured by fluorescence anisotropy assay in the absence (a) or presence of 100 µM of molecular glue R1 (b). Symbols represent the mean of a technical triplicate, with the error bars partly obscured by the symbols. The line shows a fit using a four-parameter logistic model to determine K_D or K_D^{app} values (shown in Table S5).

Table S5. K_D or K_D^{app} values obtained from the titration of 14-3-3γ to 100 nM of FAM-labeled ERRγ peptide (Figure S15) at the 24 h timepoint, in the absence (DMSO) or presence of 100 µM of compound **R1**. Data obtained from a fit using a four-parameter logistic model to determine K_D or K_D^{app} values as 95% confidence interval. The means were used for the calculation of the stabilization factor.

pH	DMSO: K_D at 24 h (µM)	Compound R1: K_D^{app} at 24 h (µM)	Stabilization factor
**6.5**	3.2-3.6	1.2-1.3	3
**7.0**	4.2-4.6	0.53-0.59	8
**7.5**	3.5-4.2	0.09-0.10	40
**8.0**	4.3-8.6	0.054-0.061	103
**8.5**	6.9-9.2	0.049-0.055	152

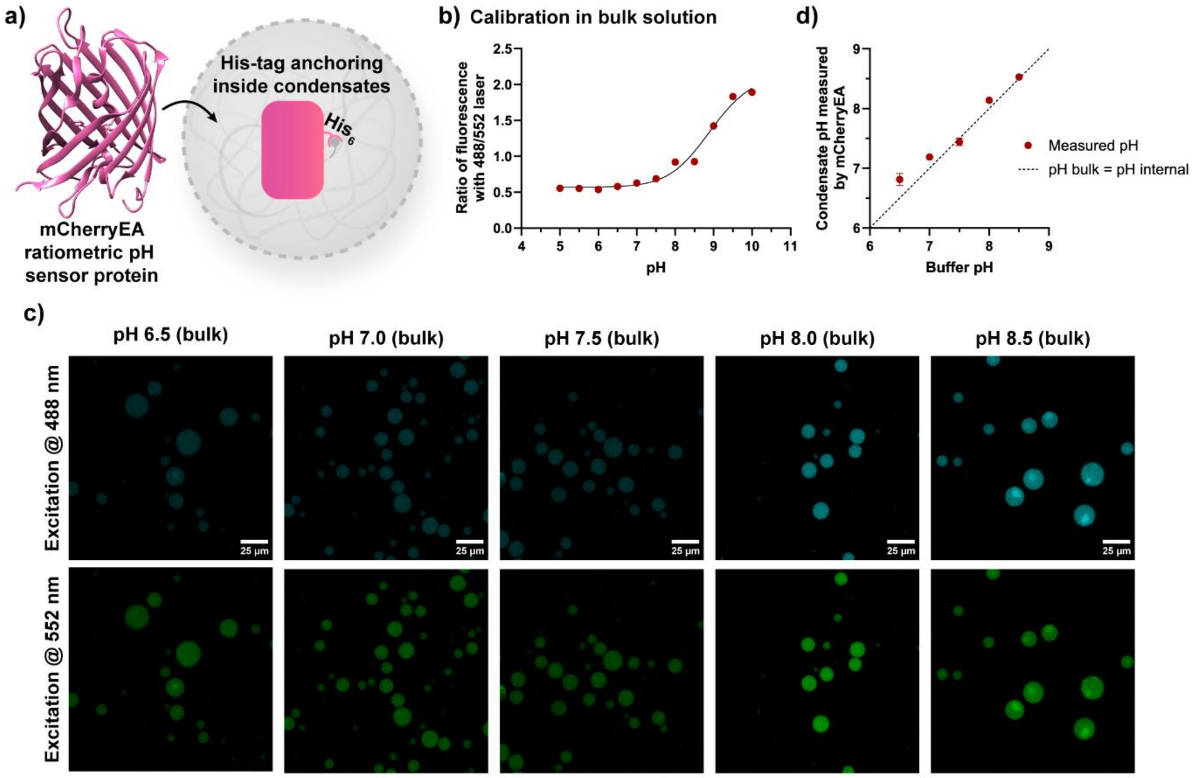

Figure S16. a) Schematic overview of mCherryEA structure and uptake into synthetic condensates. The structure shown is that of the non-mutated mCherry, PDB: 2H5Q.[13] b) Calibration of mCherryEA in bulk solution (50 µM) using confocal microscopy and calculation of the ratio of excitation at 488 nm or 552 nm, with collection of emission between 562-700 nm in both settings. c) Confocal micrographs of condensates prepared at different pH (bulk pH values shown), with 500 nM of mCherryEA. Excitation was performed at 488 nm or 552 nm, with collection of emission between 562-700 nm in both settings. d) Plot of the determined local pH values in the condensates relative to the buffer pH (bulk), as determined from the samples in panel c and the calibration curve.

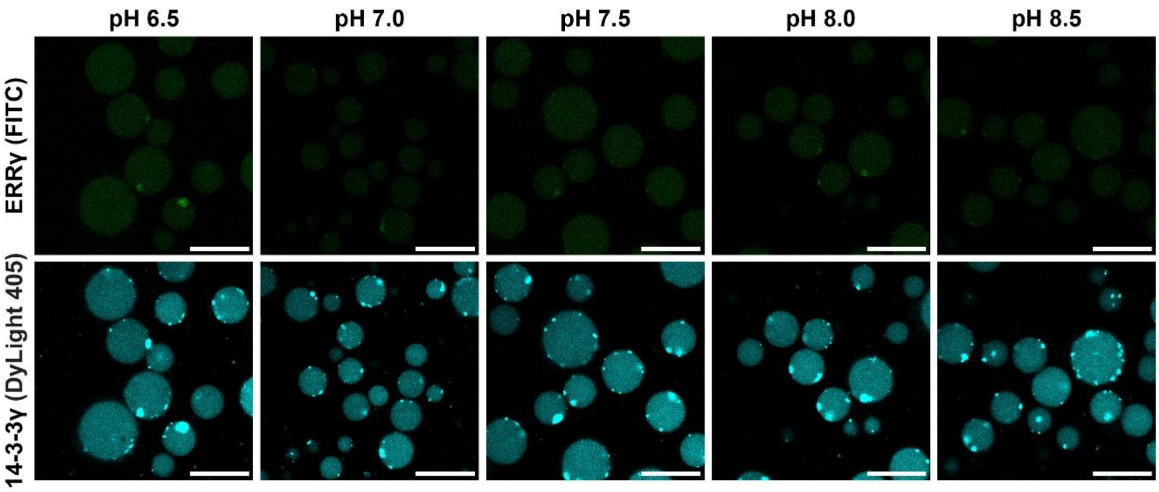

Figure S17. The binary 14-3-3/ERRy interaction is mostly non-sensitive to pH. Confocal micrographs of 14-3-3γ-loaded condensates supplied with FITC-ERRγ (100 nM) in the absence of compound **R1** (DMSO control) at various pH values. Samples were incubated overnight. Scale bar: 25 µm.

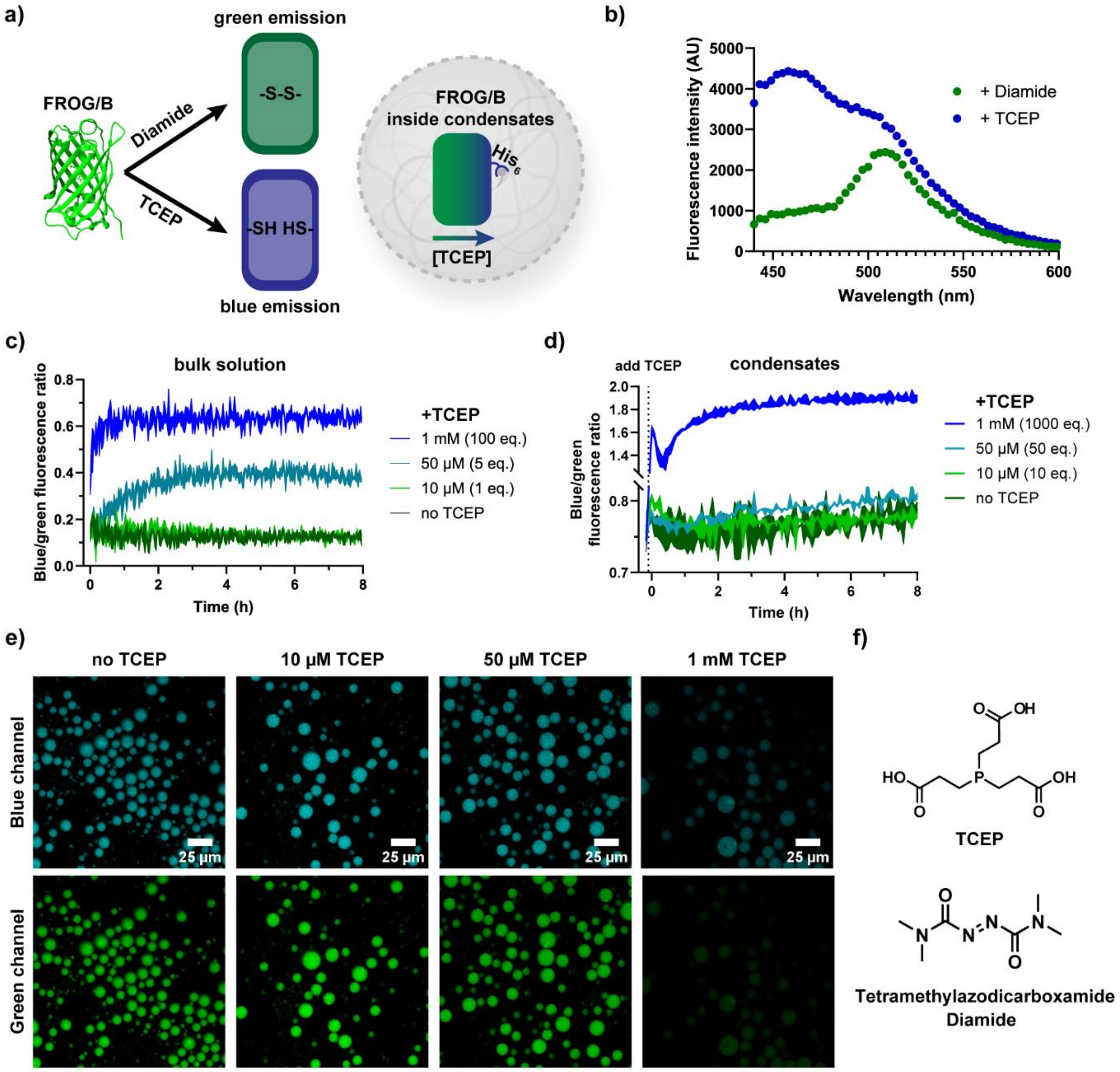

Figure S18. a) Schematic overview of FROG/B protein sensor used for redox sensing inside condensates. The FROG/B protein[15] contains a cysteine pair near the active site that influences the emission wavelength of the chromophore depending on the oxidation state. In the oxidized disulfide-containing state, the protein mainly emits green light upon excitation with 405 nm. In the reduced free thiol state, the protein mainly emits blue light upon excitation. b) Fluorescence spectroscopy of FROG/B (10 μM) in bulk solution, containing 9 eq. of the oxidizing agent diamide or the reducing agent TCEP, incubated for 20 minutes. Excitation was performed at 400 ± 20 nm. c) Reduction of oxidized FROG/B (10 μM) in bulk solution as measured by fluorescence spectroscopy. Different concentrations of TCEP were added and the blue/green fluorescence ratio was measured over time. Excitation was performed at 400 ± 20 nm. Blue emission was collected at 450 ± 20 nm and green emission at 510 ± 20 nm. N = 3, with data shown as a shaded area of the mean ± standard deviation. d, e) Reduction of oxidized FROG/B (1 μM) in condensates as measured by confocal microscopy upon excitation with 405 nm and collection of the emission at 410-480 nm (blue) and 480-550 nm (green). The blue/green ratio is shown as a shaded area of the mean ± standard deviation (d), quantified from micrographs at 2 distinct positions in the samples (e). f) Structure of the reducing agent TCEP and the oxidizing agent diamide.

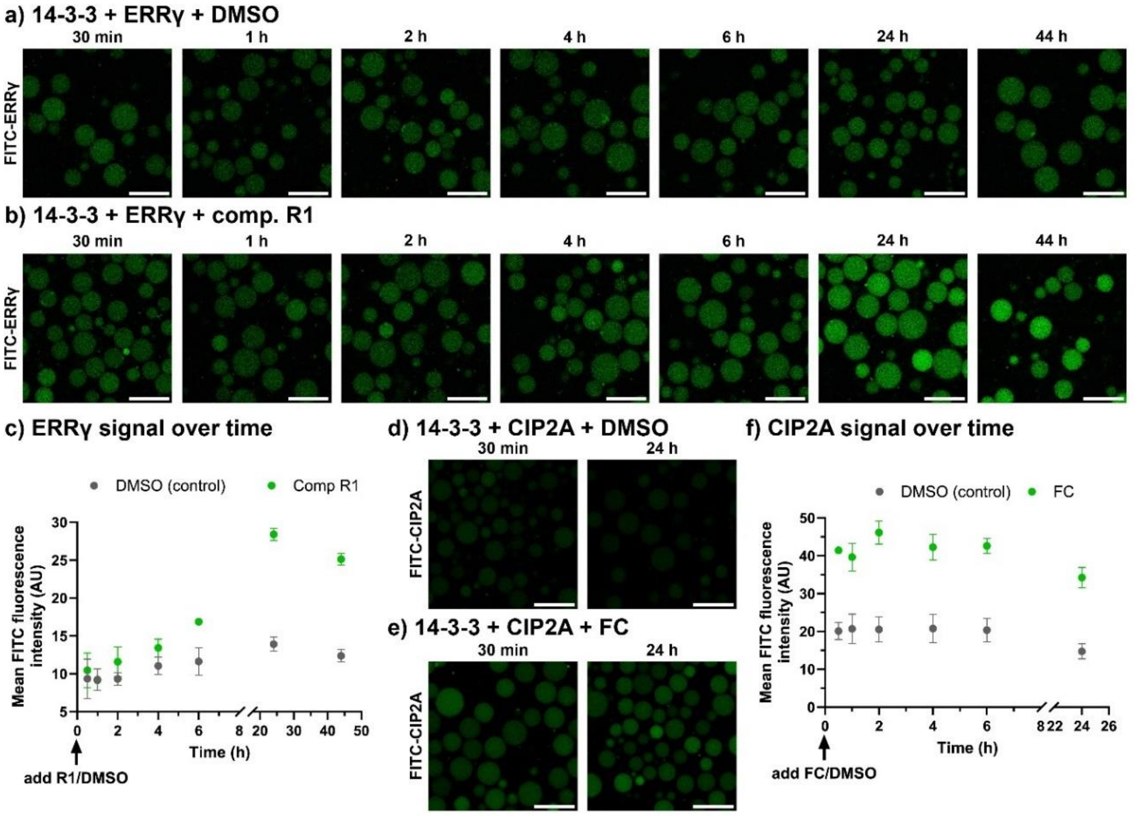

Figure S19. Compound **R1** reacts over time to yield ERRy recruitment, whereas the noncovalent molecular glue FC shows no change over time. a,b) Confocal micrographs of 14-3-3γ-loaded condensates supplied with FITC-ERRγ (100 nM) in the absence (a) or presence (b) of 100 µM of compound **R1**, measured over time. Scale bar: 25 µm. Only the FITC channel is shown and it was digitally adjusted for visibility. c) Quantification of the samples shown in panels a and b, shown as mean ± standard deviation, N=2 (compound **R1**) or N=3 (DMSO) independent condensate samples with quantification of ≥ 67 condensates each. d,e) Confocal micrographs of 14-3-3σ-loaded condensates supplied with FITC-CIP2A (100 nM) in the absence (d) or presence (e) of 100 µM of FC, measured over time. Scale bar: 25 µm. Only the FITC channel is shown. f) Quantification of the samples shown in panel c, shown as mean ± standard deviation, N=3 independent condensate samples with quantification of ≥ 67 condensates each.

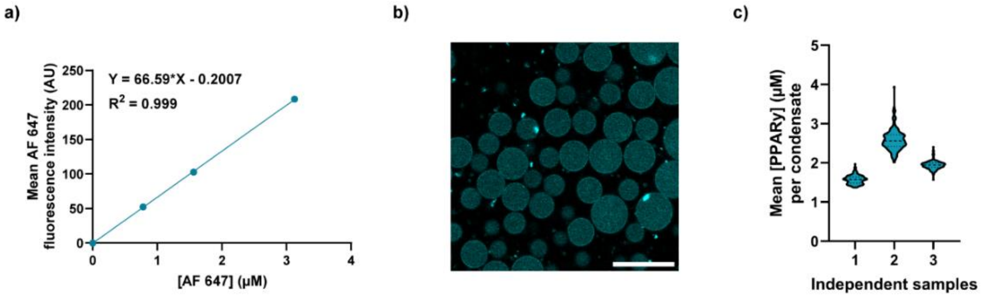

Figure S20. Measurement of the local concentration of PPARγ in the condensates. a) Calibration curve of AF 647 in condensate buffer by confocal microscopy, using the same settings used for the condensate samples. The formula was obtained by linear regression. b) Confocal micrograph of condensates loaded with AF 647-labeled PPARγ (400 nM bulk concentration). c) Quantification of micrographs of independent condensate samples containing 400 nM of AF 647-labeled PPARγ, revealing the distribution of local concentrations of PPARγ in the condensates. The degree of dye labeling of the protein was determined to be 0.38. N ≥ 130 condensates analyzed for each sample.

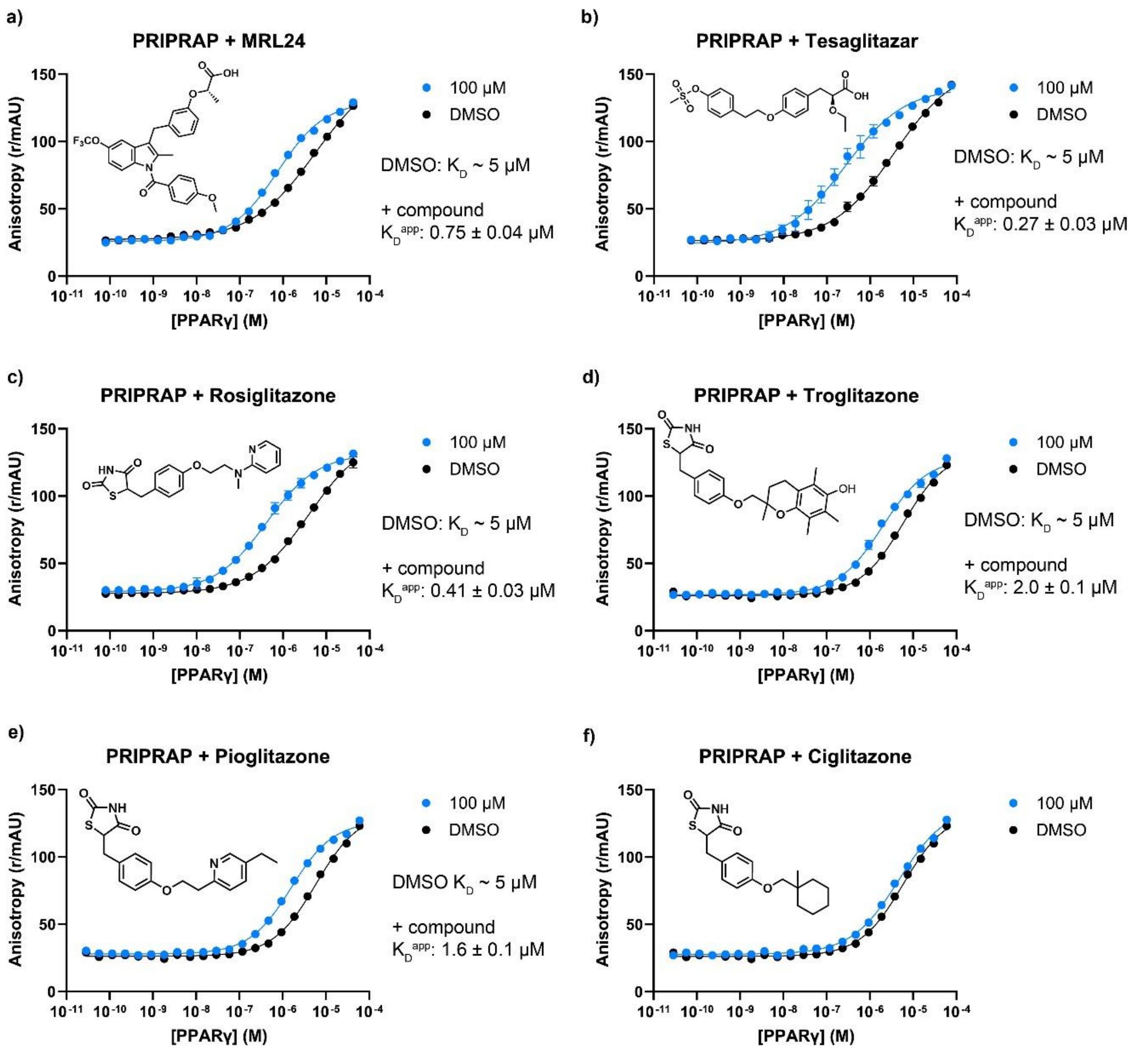

Figure S21. Protein titrations of PPARγ to the PRIPRAP peptide (10 nM), as measured by fluorescence anisotropy assay in the presence of DMSO (control) or various PPAR ligands (a-f) in bulk solution. Symbols represent the mean of a technical duplicate or triplicate (in the case of troglitazone, pioglitazone, and ciglitazone), with the error bars partly obscured by the symbols. Lines show fits by a 4-parameter logistic model. The resulting K_D or K_D^{app} obtained from the fit is shown as mean with the standard error.

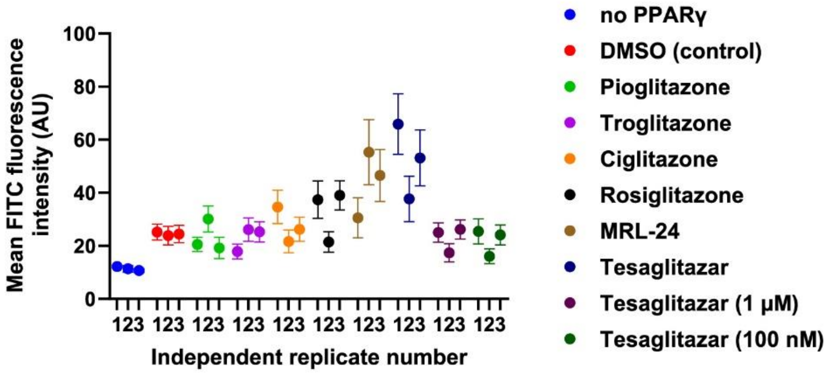

Figure S22. Quantification of PRIPRAP signal from condensates in the micrographs shown in Figure 6, plotted per individual replicate. The numbers represent the independently formulated coacervate batches.

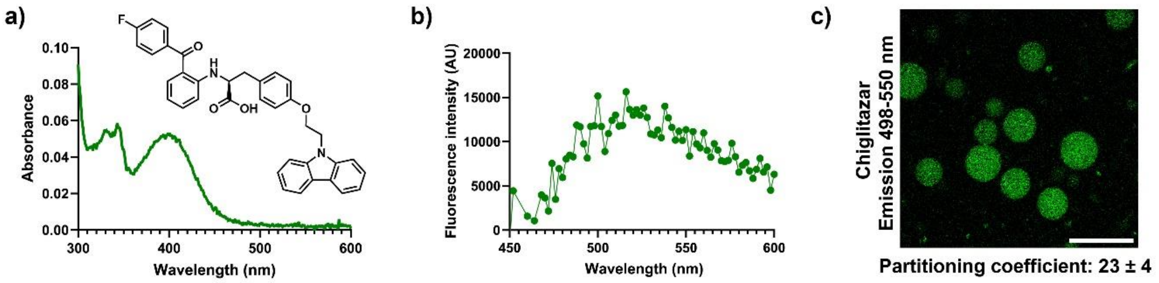

Figure S23. a) Absorbance spectrum of chiglitazar (10 µM) in bulk solution, with the structure shown as inset. b) Fluorescence spectrum of chiglitazar (10 µM) in bulk solution upon excitation with 400 ± 20 nm. c) Confocal micrograph of condensates supplemented with 10 µM of chiglitazar, excited at 405 nm and measured at 498-550 nm. The relative intensity inside the condensates versus in dilute solution was used to calculate the partitioning coefficient. N = 3 technical replicates from the same sample, data presented as mean ± standard deviation.

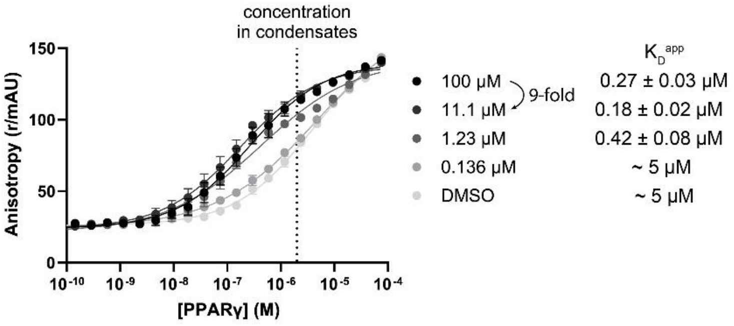

Figure S24. Protein titrations of PPARγ to the PRIPRAP peptide (10 nM), as measured by fluorescence anisotropy assay in bulk solution. The titration was carried out in the presence of DMSO (control) or various concentrations of tesaglitazar. Symbols represent the mean of a technical duplicate, with the error bars partly obscured by the symbols. Lines show fits by a 4-parameter logistic model. The resulting K_D^{app} or K_D (DMSO) obtained from the fit is shown as mean with the standard error.

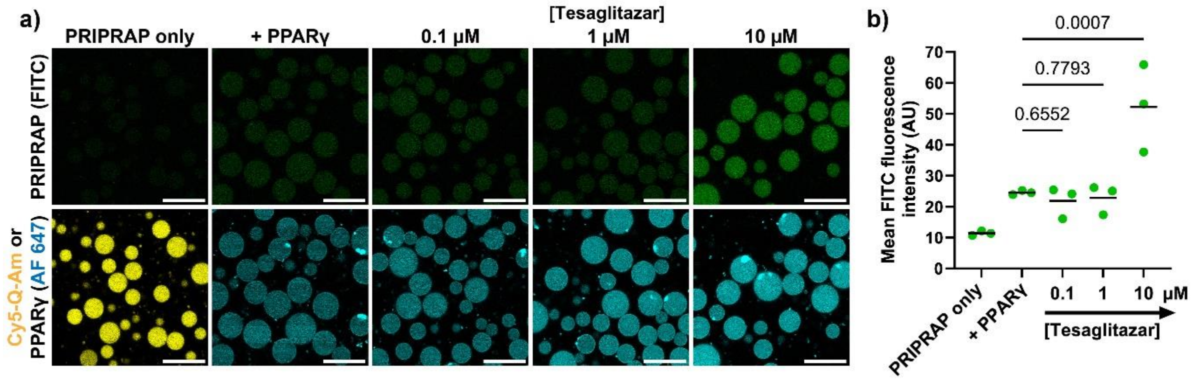

Figure S25. a) Confocal micrographs of tesaglitazar-dependent uptake of PRIPRAP coregulator (100 nM) in condensates loaded with PPARγ (400 nM bulk concentration). Scale bar: 25 µm. b) Quantification of PRIPRAP (FITC) from micrographs. Statistical analysis was performed by one-way ANOVA with Tukey's test with correction for multiple comparisons, with N = 3 independent condensate samples. P values are shown above the comparison. The lines show the means, and the symbols show the individual measurements.

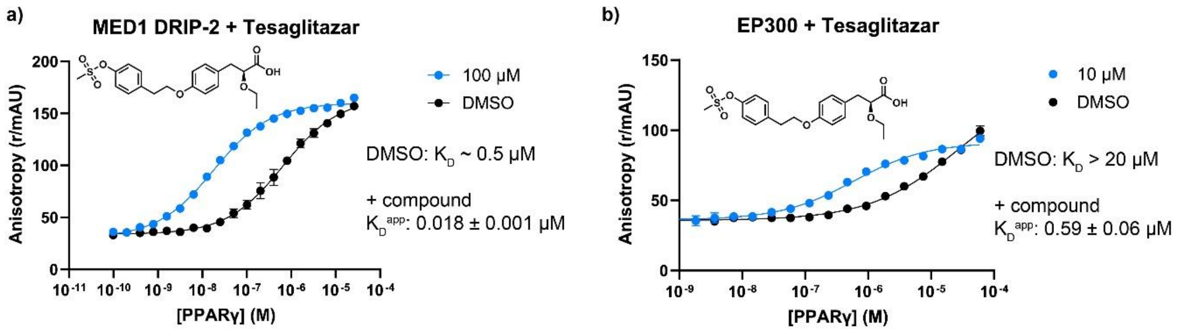

Figure S26. a) Protein titrations of PPARγ to the MED1 DRIP-2 peptide (10 nM), as measured by fluorescence anisotropy assay in the presence of DMSO (control) or 100 µM of Tesaglitazar in bulk solution. Symbols represent the mean of a technical duplicate, with the error bars partly obscured by the symbols. Lines show fits by a 4-parameter logistic model. The resulting K_D^{app} or K_D values obtained from the fit are shown as mean with the standard error. b) Protein titrations of PPARγ to the EP300 peptide (10 nM), as measured by fluorescence anisotropy assay in the presence of DMSO (control) or Tesaglitazar (10 µM) in bulk solution. Symbols represent the mean of a technical duplicate, with the error bars partly obscured by the symbols. Lines show fits by a 4-parameter logistic model. The resulting K_D^{app} or K_D values obtained from the fit are shown as mean with the standard error.

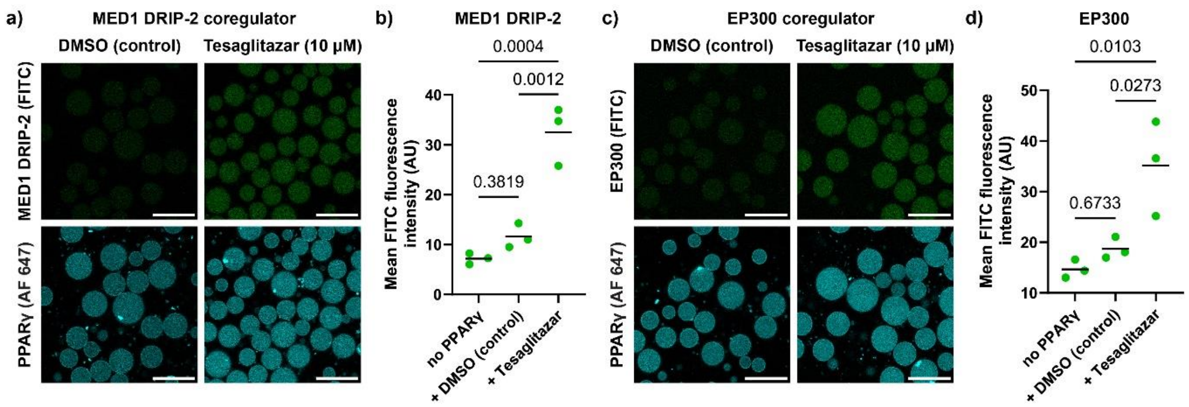

Figure S27. a) Confocal micrographs of ligand-dependent uptake of MED1 DRIP-2 coregulator. Scale bar: 25 µm. b) Quantification of MED1 DRIP-2 from micrographs. Statistical analysis was performed by one-way ANOVA with Tukey's test with correction for multiple comparisons, with N = 3 independent condensate samples. P values are shown above the comparison. The lines show the means, and the symbols show the individual measurements. c) Confocal micrographs of ligand-dependent uptake of EP300 coregulator. Scale bar: 25 µm. d) Quantification of EP300 from micrographs. Statistical analysis was performed by one-way ANOVA with Tukey's test with correction for multiple comparisons, with N = 3 independent condensate samples. P values are shown above the comparison. The lines show the means, and the symbols show the individual measurements.

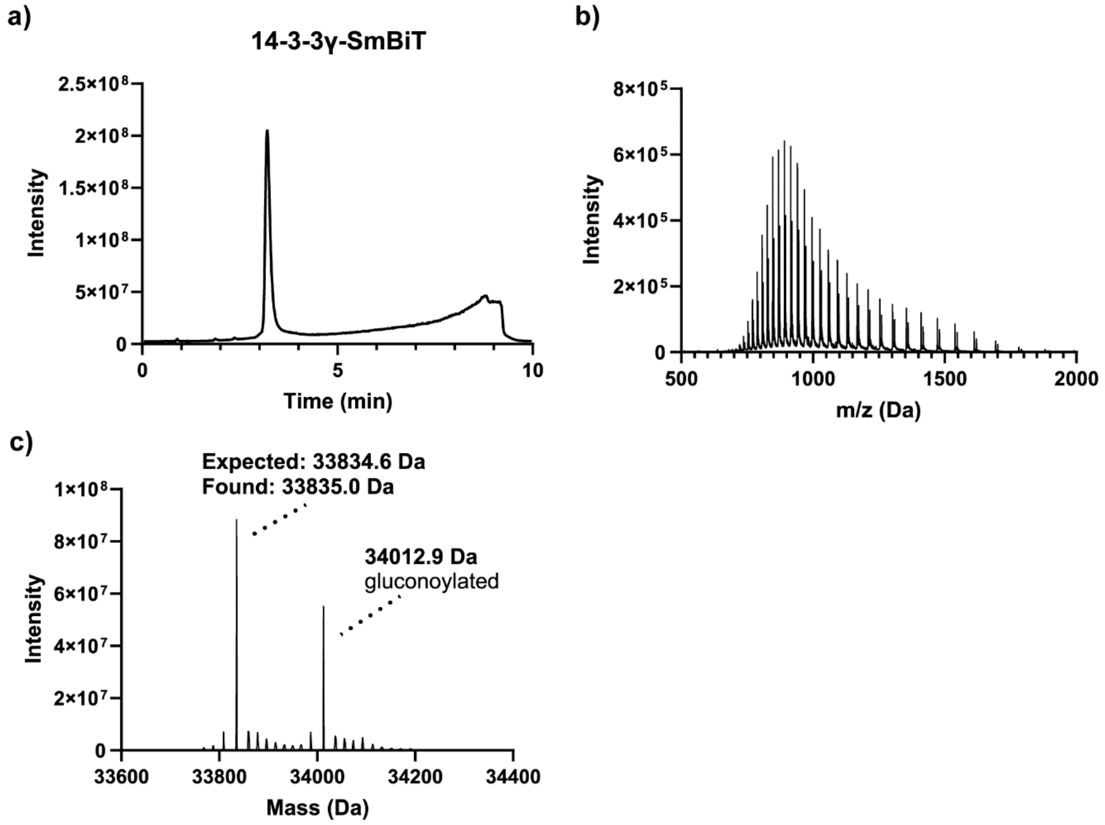

Figure S28. a) LC-MS Q-ToF chromatogram, b) m/z spectrum, and c) deconvoluted mass of 14-3-3γ-SmBiT. Expected mass for 14-3-3γ-SmBiT: 33834.6 Da (after N-terminal Met excision). Found masses: 33835.0 Da and 34012.9 Da (gluconoylated version of the former species).[16]

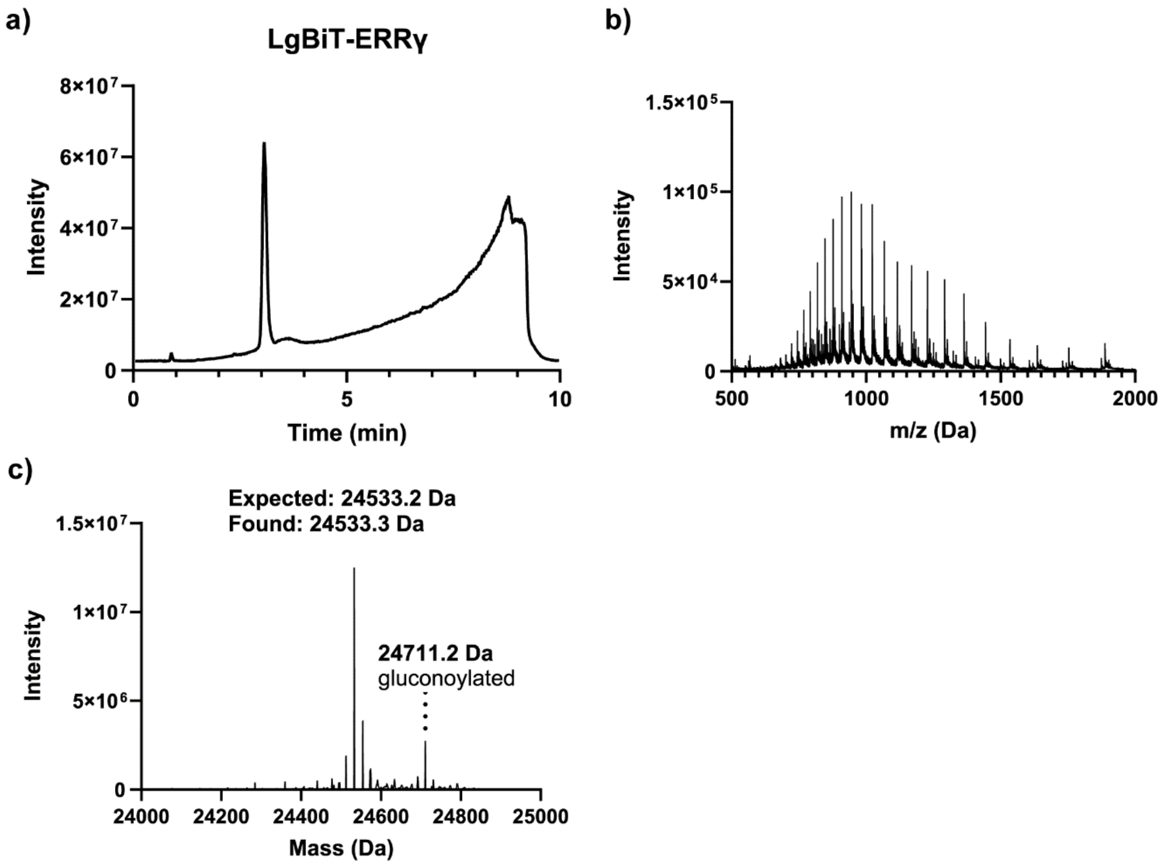

Figure S29. a) LC-MS Q-ToF chromatogram, b) m/z spectrum, and c) deconvoluted mass of LgBiT-ERRγ. Expected mass for LgBiT-ERRγ: 24533.2 Da (after N-terminal Met excision). Found masses: 24533.3 Da and 24711.2 Da (gluconoylated version of the former species).[16]

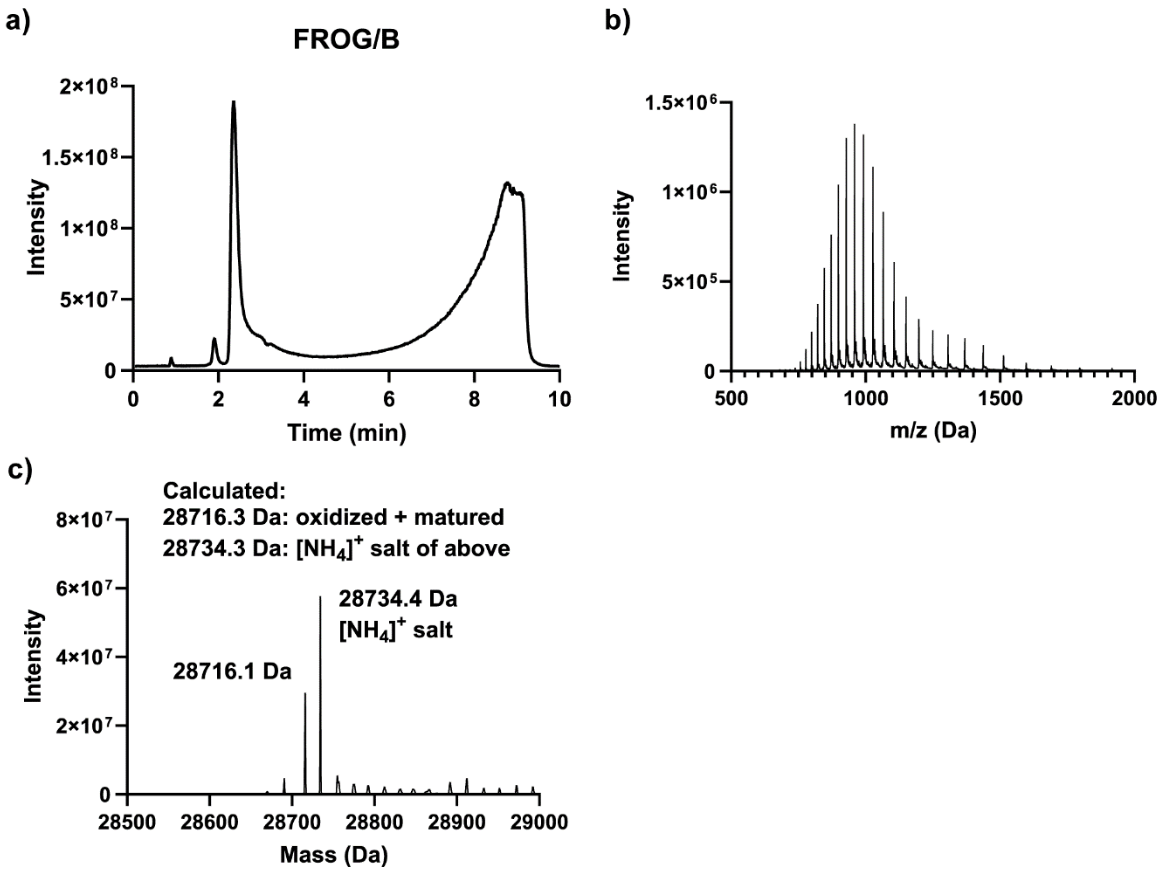

Figure S30. a) LC-MS Q-ToF chromatogram, b) m/z spectrum, and c) deconvoluted mass of FROG/B. Expected mass for FROG/B: 28716.3 Da (after N-terminal Met excision, oxidation of disulfides and maturation of chromophore). Found masses: 28716.1 Da (N-terminal Met excision, oxidation of disulfides and maturation of chromophore) and 28734.4 Da (NH_4^+ salt of the former species).

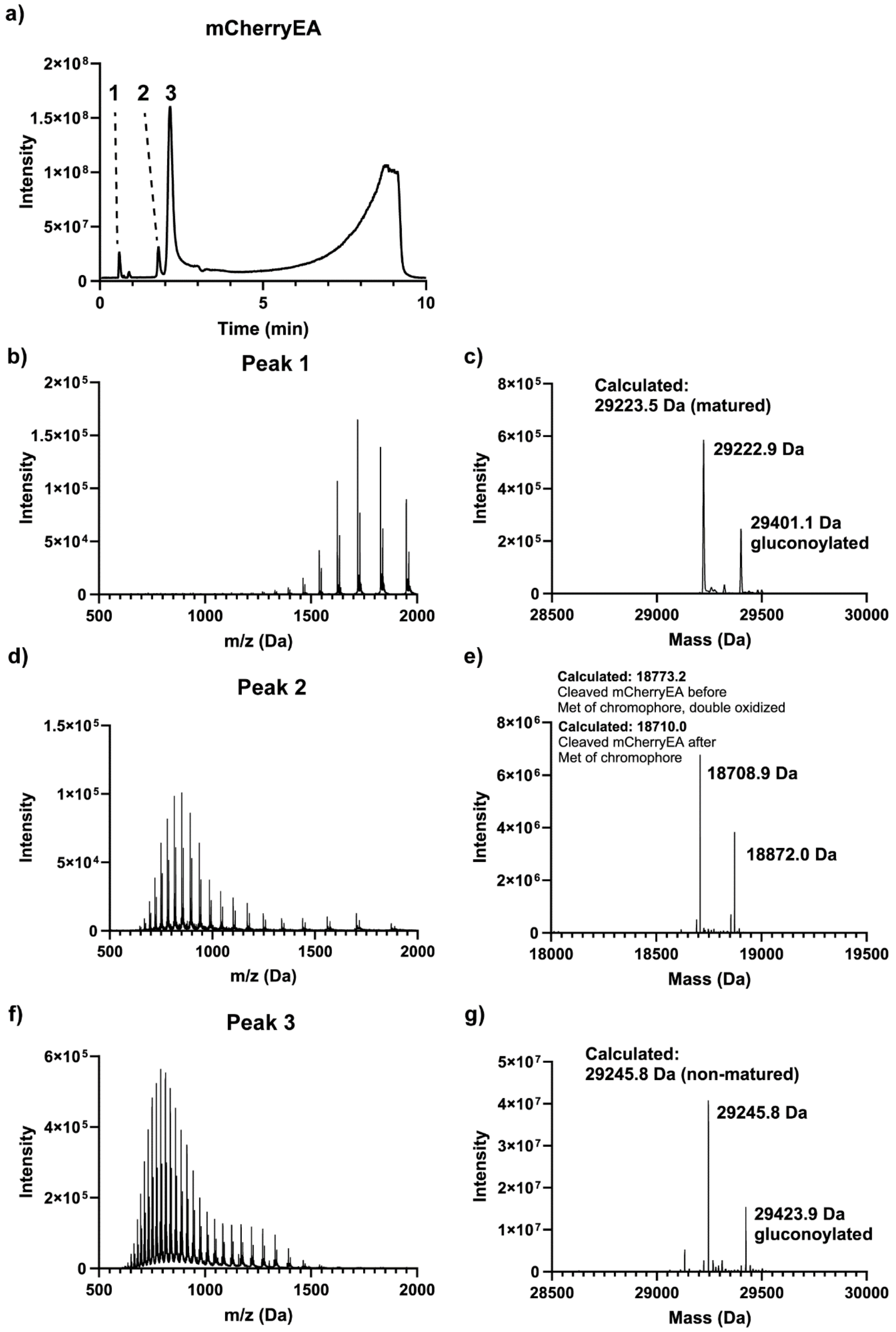

Figure S31. a) LC-MS Q-ToF chromatogram of mCherryEA. b) m/z spectrum, and c) deconvoluted mass of peak 1 in the chromatogram, corresponding to the matured mCherryEA with N-terminal methionine excision. Expected mass for matured mCherryEA: 29223.5 Da. Found masses: 29222.9 Da and 29401.1 Da (gluconoylated version of the former species).[16] d) m/z spectrum, and e) deconvoluted mass of peak 2 in the chromatogram, corresponding to cleavage products of mCherryEA before or after the Met residue of the chromophore (Met94). f) m/z spectrum, and g) deconvoluted mass of peak 3 in the chromatogram, corresponding to non-matured mCherryEA. Expected mass for non-matured mCherryEA: 29245.8 Da. Found masses: 29245.8 Da and 29423.9 Da (gluconoylated version of the former species).[16]

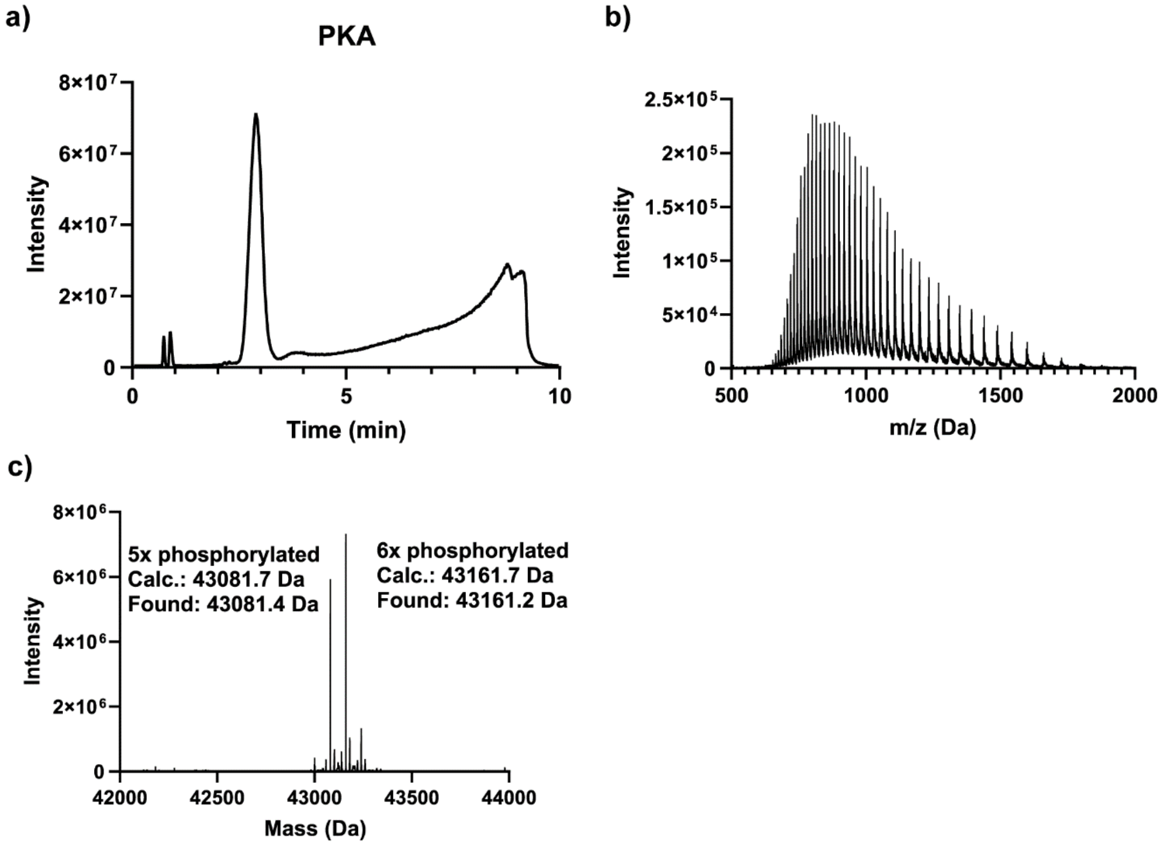

Figure S32. a) LC-MS Q-ToF chromatogram, b) m/z spectrum, and c) deconvoluted mass of PKA (catalytic subunit). Calculated masses for PKA: 43081.7 Da (after N-terminal Met excision and 5x autophosphorylation) and 43161.7 Da (after N-terminal Met excision and 6x autophosphorylation). Found masses: 43081.4 Da and 43161.2 Da.

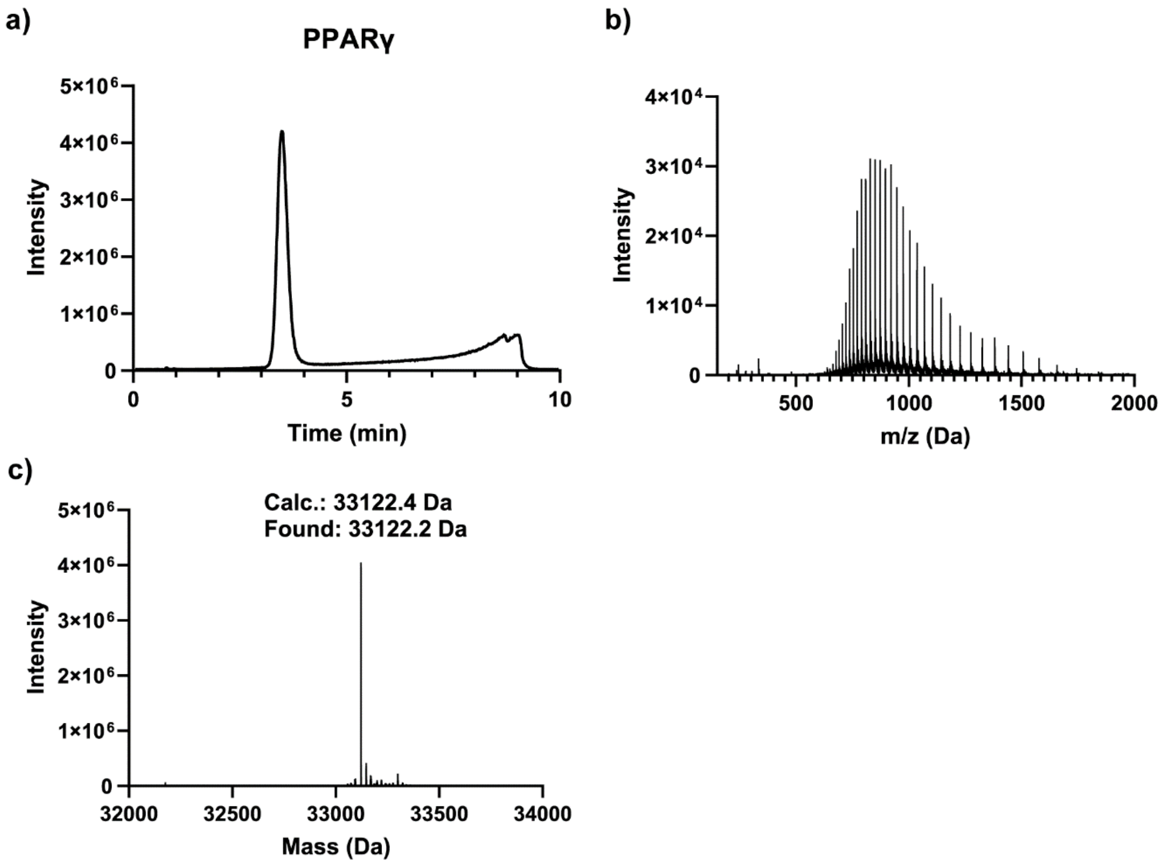

Figure S33. a) LC-MS Q-ToF chromatogram, b) m/z spectrum, and c) deconvoluted mass of PPARγ (ligand binding domain). Calculated masses for PPARγ: 33122.4 Da (after N-terminal Met excision. Found mass: 33122.2 Da.

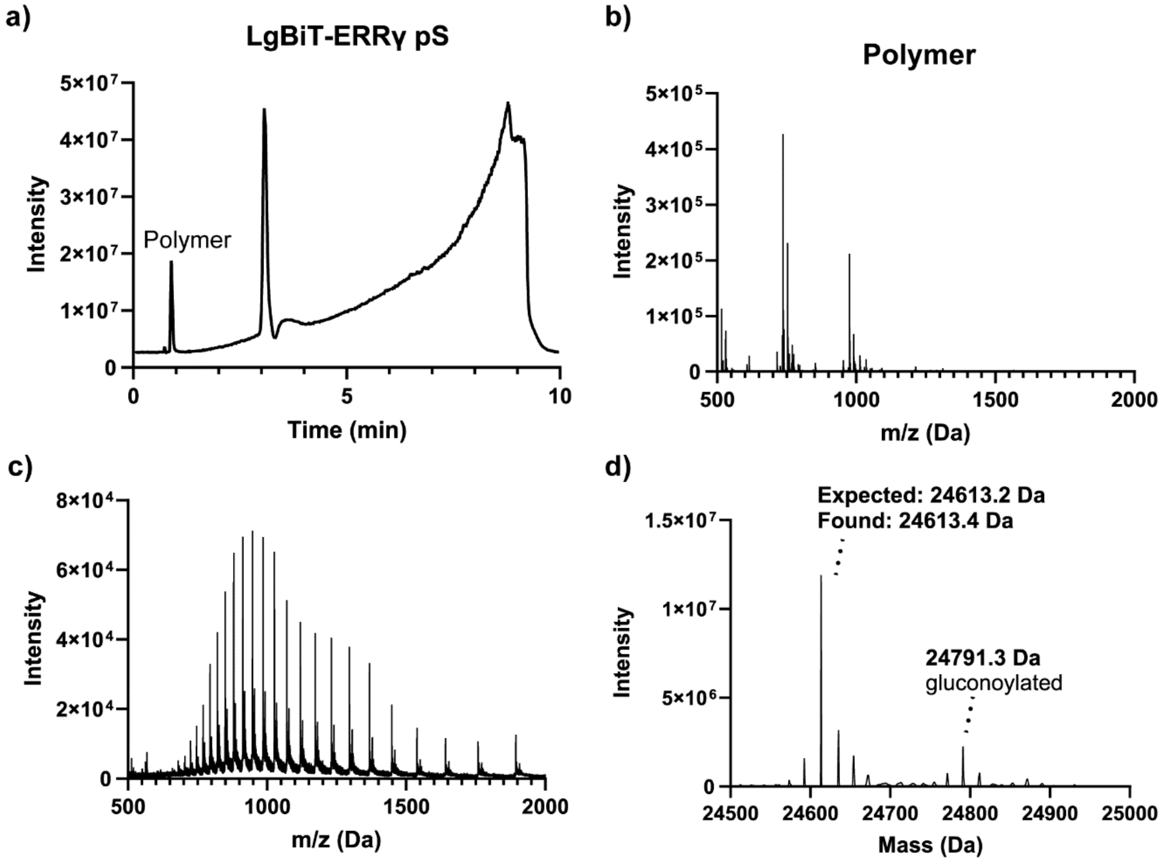

Figure S34. a) LC-MS Q-ToF chromatogram, b) m/z spectrum, and c) deconvoluted mass of LgBiT-ERRγ pS (phosphorylated). Expected mass for LgBiT-ERRγ pS: 24613.2 Da (after N-terminal Met excision and single phosphorylation). Found masses: 24613.4 Da and 24791.3 Da (gluconoylated version of the former species).[16]

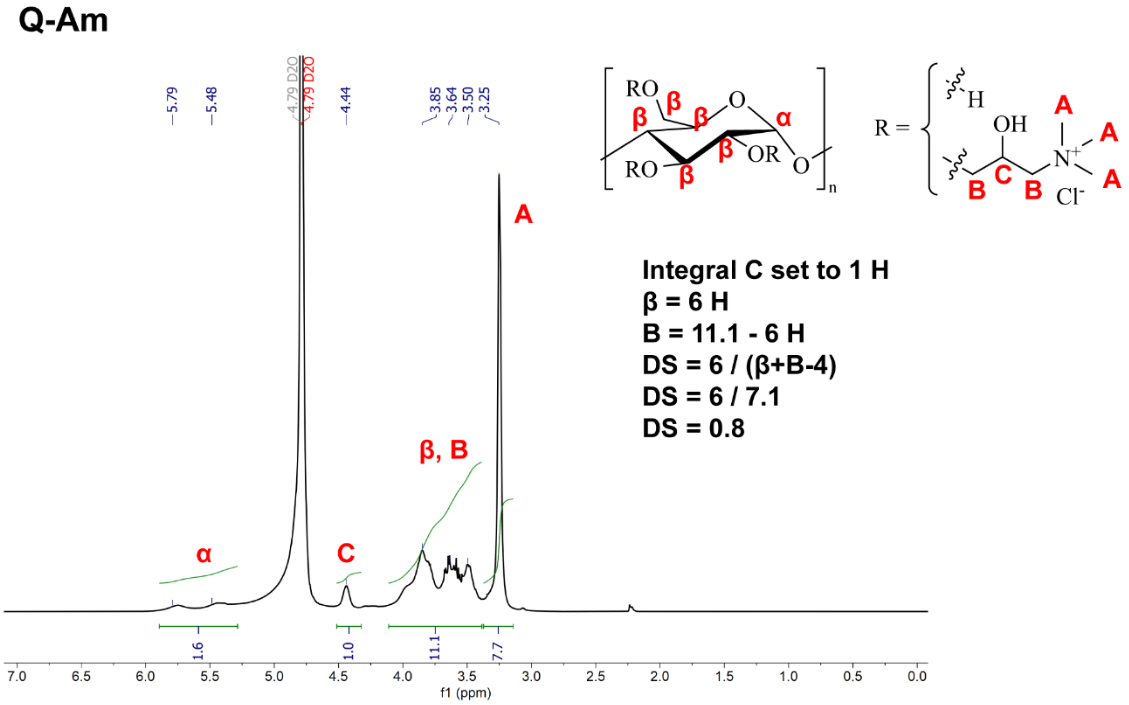

Figure S35. ¹H NMR spectrum of the main batch of Q-Am in D_2O, with calculation of the degree of substitution.

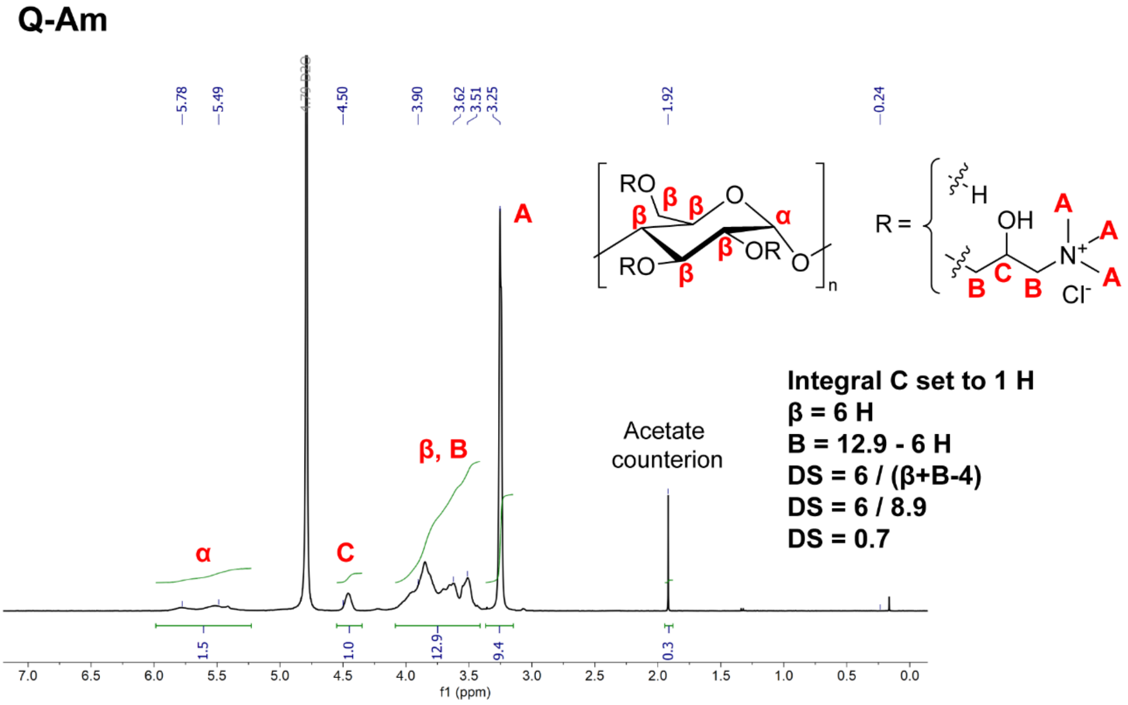

Figure S36. ¹H NMR spectrum (400 MHz) of Q-Am used for the LC-MS experiment, in D_2O, with calculation of the degree of substitution.

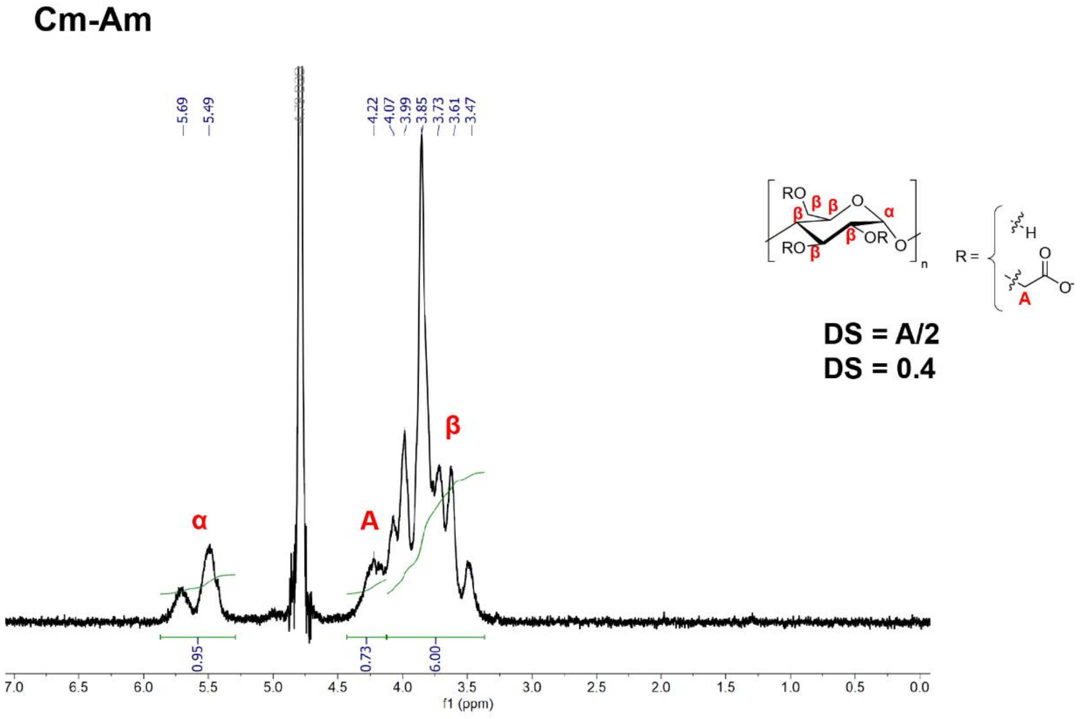

Figure S37. ^{1}H NMR spectrum (400 MHz) of Cm-Am in D_2O, with calculation of the degree of substitution.

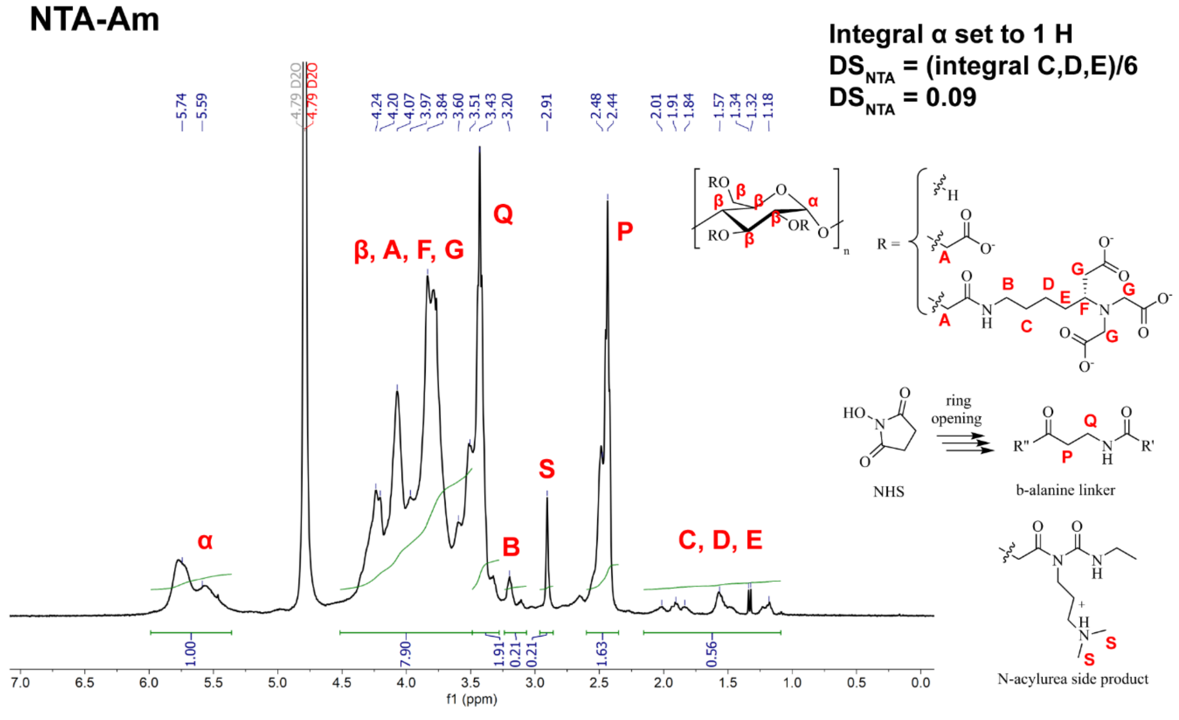

Figure S38. ^{1}H NMR spectrum (400 MHz) of NTA-Am in D_2O, with calculation of the degree of substitution. Signals corresponding to common EDC/NHS side reactions were observed: β-alanine formation and N-acylurea formation.[6]

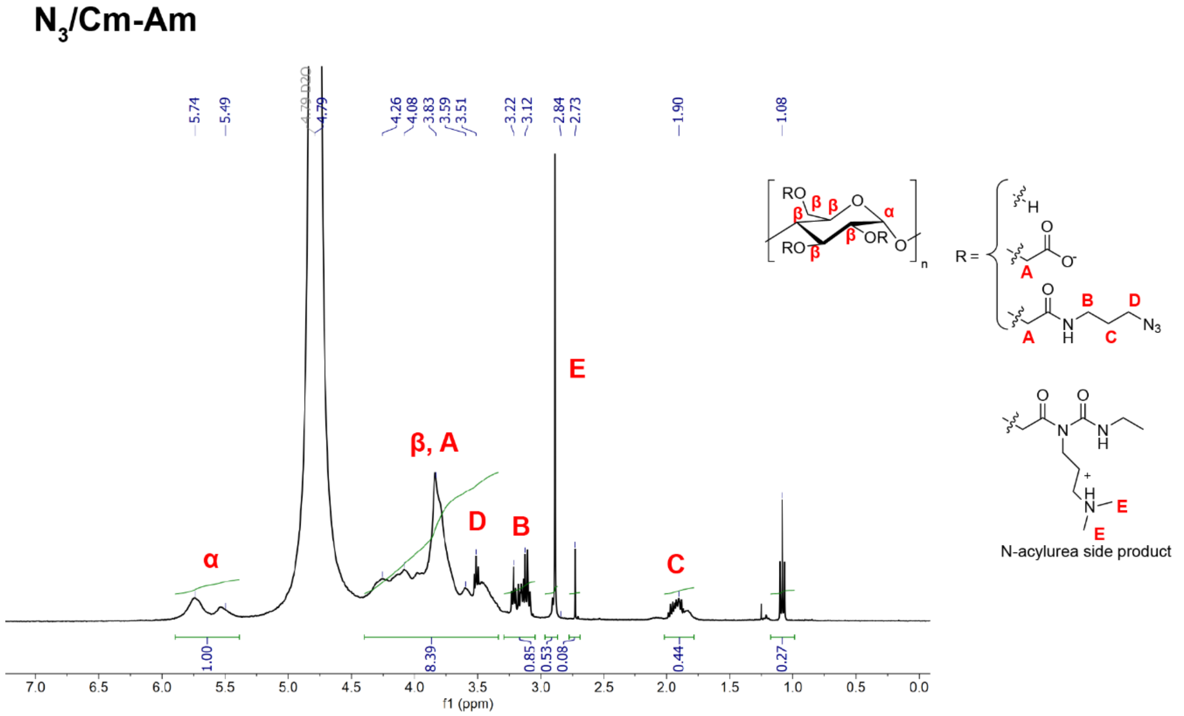

Figure S39. ^{1}H NMR spectrum (400 MHz) of N_3/Cm-Am in D_2O. Signals corresponding to common EDC/NHS side reactions were observed: N-acylurea formation.[6]

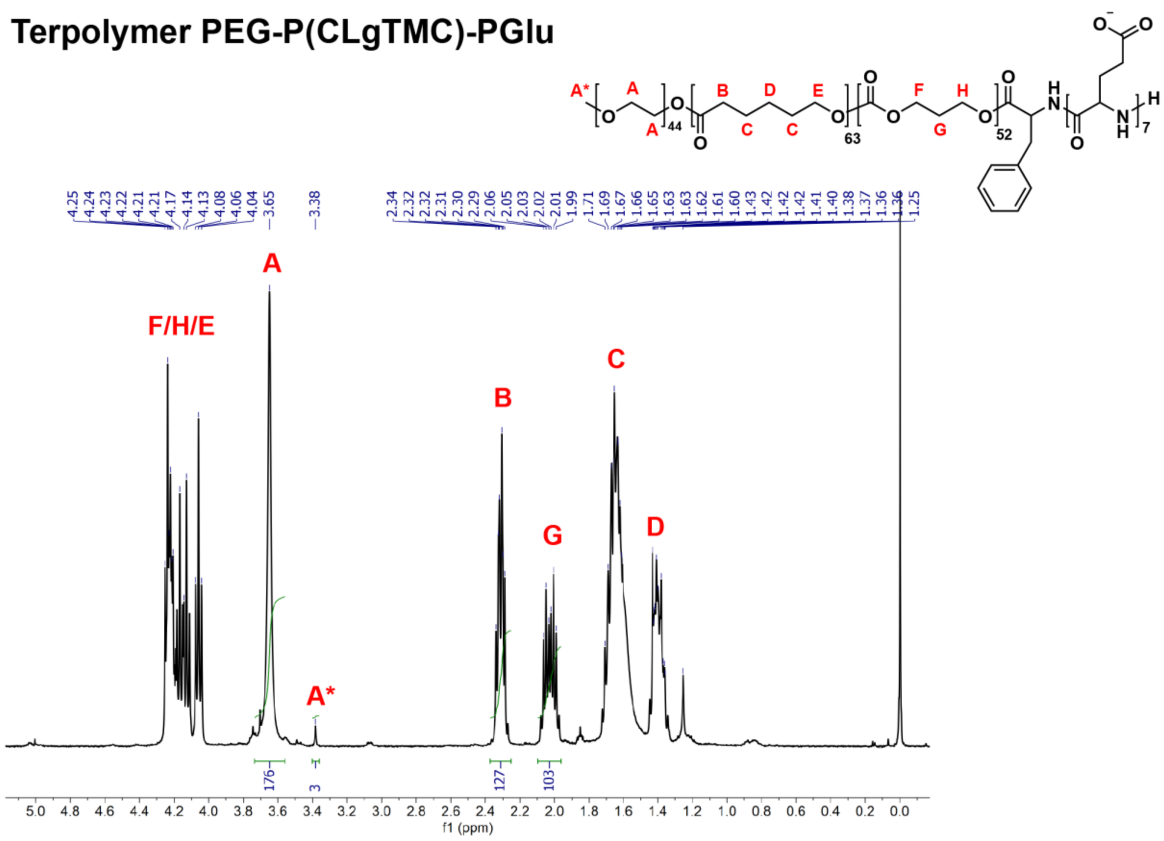

Figure S40. ^{1}H NMR spectrum (400 MHz) of terpolymer in $CDCl_3$.

References

1. Andrei, S. A. *et al.* Rationally Designed Semisynthetic Natural Product Analogues for Stabilization of 14-3-3 Protein–Protein Interactions. *Angew. Chemie - Int. Ed.* **57**, 13470–13474 (2018).

2. De Vink, P. J. *et al.* Cooperativity basis for small-molecule stabilization of protein-protein interactions. *Chem. Sci.* **10**, 2869–2874 (2019).

3. Somsen, B. A. *et al.* Reversible Dual-Covalent Molecular Locking of the 14-3-3/ERRγ Protein-Protein Interaction as a Molecular Glue Drug Discovery Approach. *J. Am. Chem. Soc.* **145**, 6741–6752 (2023).

4. Mason, A. F., Buddingh, B. C., Williams, D. S. & van Hest, J. C. M. Hierarchical Self-Assembly of a Copolymer-Stabilized Coacervate Protocell. *J. Am. Chem. Soc.* **139**, 17309–17312 (2017).

5. Altenburg, W. J. *et al.* Programmed spatial organization of biomacromolecules into discrete, coacervate-based protocells. *Nat. Commun.* **11**, 6282 (2020).

6. Totaro, K. A. *et al.* Systematic Investigation of EDC/sNHS-Mediated Bioconjugation Reactions for Carboxylated Peptide Substrates. *Bioconjug. Chem.* **27**, 994–1004 (2016).

7. Mason, A. F., Altenburg, W. J., Song, S., van Stevendaal, M. & van Hest, J. C. M. Terpolymer-stabilized complex coacervates: A robust and versatile synthetic cell platform. *Methods Enzymol.* **646**, 51–82 (2021).

8. Pettersen, E. F. *et al.* UCSF Chimera - A visualization system for exploratory research and analysis. *J. Comput. Chem.* **25**, 1605–1612 (2004).

9. Boston, P. F., Jackson, P. & Thompson, R. J. Human 14-3-3 Protein: Radioimmunoassay, Tissue Distribution, and Cerebrospinal Fluid Levels in Patients with Neurological Disorders. *J. Neurochem.* **38**, 1475–1482 (1982).

10. Ellis, R. J. Macromolecular crowding: Obvious but underappreciated. *Trends Biochem. Sci.* **26**, 597–604 (2001).

11. Brink, H. J. *et al.* Fusicoccin-A Targets Cancerous Inhibitor of Protein Phosphatase 2A by Stabilizing a C-Terminal Interaction with 14-3-3. *ACS Chem. Biol.* **17**, 2972–2978 (2022).

12. Sluchanko, N. N. *et al.* Structural Basis for the Interaction of a Human Small Heat Shock Protein with the 14-3-3 Universal Signaling Regulator. *Structure* **25**, 305–316 (2017).

13. Shu, X., Shaner, N. C., Yarbrough, C. A., Tsien, R. Y. & Remington, S. J. Novel chromophores and buried charges control color in mFruits. *Biochemistry* **45**, 9639–9647 (2006).

14. Pédelacq, J. D., Cabantous, S., Tran, T., Terwilliger, T. C. & Waldo, G. S. Engineering and characterization of a superfolder green fluorescent protein. *Nat. Biotechnol.* **24**, 79–88 (2006).

15. Sugiura, K., Mihara, S., Fu, N. & Hisabori, T. Real-time monitoring of the in vivo redox state transition using the ratiometric redox state sensor protein FROG/B. *Proc. Natl. Acad. Sci. U. S. A.* **117**, 16019–16026 (2020).

16. Geoghegan, K. F. *et al.* Spontaneous α-N-6-phosphogluconoylation of a 'His tag' in Escherichia coli: The cause of extra mass of 258 or 178 Da in fusion proteins. *Anal. Biochem.* **267**, 169–184 (1999).
